# Comparative genomics of the *Komagataeibacter* strains—Efficient bionanocellulose producers

**DOI:** 10.1002/mbo3.731

**Published:** 2018-10-26

**Authors:** Małgorzata Ryngajłło, Katarzyna Kubiak, Marzena Jędrzejczak‐Krzepkowska, Paulina Jacek, Stanisław Bielecki

**Affiliations:** ^1^ Institute of Technical Biochemistry Lodz University of Technology Lodz Poland

**Keywords:** bacterial cellulose, c‐di‐GMP network, comparative genomics, exopolysaccharides, genome flexibility, *Komagataeibacter*

## Abstract

*Komagataeibacter* species are well‐recognized bionanocellulose (BNC) producers. This bacterial genus, formerly assigned to *Gluconacetobacter*, is known for its phenotypic diversity manifested by strain‐dependent carbon source preference, BNC production rate, pellicle structure, and strain stability. Here, we performed a comparative study of nineteen *Komagataeibacter* genomes, three of which were newly contributed in this work. We defined the core genome of the genus, clarified phylogenetic relationships among strains, and provided genetic evidence for the distinction between the two major clades, the *K*. *xylinus* and the *K. hansenii*. We found genomic traits, which likely contribute to the phenotypic diversity between the *Komagataeibacter* strains. These features include genome flexibility, carbohydrate uptake and regulation of its metabolism, exopolysaccharides synthesis, and the c‐di‐GMP signaling network. In addition, this work provides a comprehensive functional annotation of carbohydrate metabolism pathways, such as those related to glucose, glycerol, acetan, levan, and cellulose. Findings of this multi‐genomic study expand understanding of the genetic variation within the *Komagataeibacter* genus and facilitate exploiting of its full potential for bionanocellulose production at the industrial scale.

## 1 INTRODUCTION

The *Komagataeibacter* genus has recently emerged within the family of acetic acid bacteria (AAB) and consists of fourteen species (Barja, Andrés‐Barrao, Ortega Pérez, María Cabello, & Chappuis, 2016; Duardo, Ryngajllo, Jedrzejczak‐Krzepkowska, Bielecki, & Gama, 2016; Yamada, 2014, 2016; Yamada, Yukphan, Lan Vu, et al. 2012; Yamada, Yukphan, Vu, et al. 2012). This genus was named after a Japanese microbiologist, Prof. Kazuo Komagata, in appreciation of his contribution to the systematics of AAB. These species were mostly isolated from: vinegar (*K*. *xylinus*,* K*. *europaeus*,* K*. *hansenii*,* K*. *oboediens*,* K*. *kakiaceti*,* K*. *medellinensis, K*. *maltaceti, Ga. entanii*), fruit or fruit juice (*K*. *xylinus, K*. *rhaeticus, K*. *swingsii, K*. *saccharivorans, K*. *sucrofermentans*), tea fungus beverage—Kombucha (*K*. *intermedius, K*. *rhaeticus*), or from nata de coco (*K*. *nataicola*) (Yamada, 2016). This genus, apart from being an obligate aerobe and a gram‐negative alpha‐proteobacteria, is characterized by a lack of flagellation, production of acetic acid from ethanol, growth in the presence of 0.35% acetic acid (v/v), and no synthesis of 2,5‐diketo‐d‐gluconate from d‐glucose (Yamada, 2016). *Komagataeibacter* strains are highly resistant to acetic acid and are the dominant species of submerged vinegar processes, where acidities are highly elevated (Barja et al., 2016).

The species of *Komagataeibacter* have been shown to be the exceptionally efficient cellulose producers among bacterial species (Jedrzejczak‐Krzepkowska, Kubiak, Ludwicka, & Bielecki, 2016; Lin et al., 2013; Valera, Torija, Mas, & Mateo, 2015; Valera, Poehlein, et al., 2015). Ultrapure bionanocellulose (BNC) is finding applications in biotechnology, medicine, and various industry sectors. In comparison to plant‐derived cellulose, *Komagataeibacter* produces BNC with superior mechanical strength, purity, water‐holding capacity, and biodegradability (Cacicedo et al., 2016; Gama, Dourado, & Bielecki, 2016). Cellulose, a water‐insoluble exopolysaccharide composed of β‐1,4‐glucan chains, gives the bacterial cells protection from UV radiation or desiccation (Williams & Cannon, 1989). When growing in a liquid medium, synthesis of a cellulose membrane enables retention of cells close to the medium surface, where the amount of oxygen is high (Czaja, Young, Kawecki, & Brown, 2007; Williams & Cannon, 1989). The process of bacterial cellulose synthesis has been described only from the metabolic point of view. The cellulose synthase enzyme, which consists of four subunits, was discovered and studied in *K. xylinus* (Jedrzejczak‐Krzepkowska et al., 2016; Umeda et al., 1999; Wong et al., 1990). The function of subunits A and B (BcsA and BcsB), which are conserved among taxa, is well understood and proved to be responsible for β‐glucan chain formation (Morgan et al., 2016; Römling & Galperin, 2015). However, the role of the other subunits (BcsC and BcsD), which influence the efficiency of cellulose synthesis, is still under discussion (Hu et al., 2010; Iyer, Catchmark, Brown, & Tien, 2011; Saxena, Kudlicka, Okuda, & Brown, 1994). Moreover, there have been observed diversities in the structure of cellulose synthase operon (*bcs*) among the *Komagataeibacter* species (Matsutani et al., 2015; Saxena & Brown, 1995). It has also been reported that the efficiency of cellulose synthesis, the culturing conditions (e.g., the preferred carbon source), and structural and mechanical properties of cellulose (e.g., porosity and elasticity) depend on the bacterial strain (Czaja et al., 2007; Masaoka, Ohe, & Sakota, 1993; Suwanposri, Yukphan, Yamada, & Ochaikul, 2013; Toyosaki et al., 1995; Zeng, Laromaine, & Roig, 2014). Moreover, synthesis of soluble exopolysaccharides (EPS) may also influence cellulose features (Fang & Catchmark, 2014; Ishida, Sugano, Nakai, et al., 2002; Yoshinaga, Tonouchi, & Watanabe, 1997). Several of the *Komagataeibacter* strains were reported to synthesize acetan, derivatives of acetan, or levan (Couso, Ielpi, & Dankert, 1987; Kornmann, Duboc, Marison, & von Stockar, 2003; MacCormick, Harris, Gunning, & Morris, 1993). However, types of EPS synthesized vary not only between species but also between strains (Fang & Catchmark, 2015).

One of the well‐characterized mechanisms regulating cellulose synthesis is allosteric activation of BcsA with cyclic di‐GMP (c‐di‐GMP) molecule, a universal bacterial second messenger discovered in *K. xylinus* (Römling, 2012; Ross et al., 1985, 1987). Independent research revealed important role of c‐di‐GMP regulatory role for motility, virulence, biofilm formation, and the cell cycle control (Römling & Galperin, 2015). Cellular levels of c‐di‐GMP are under control of proteins with opposite enzymatic activities: diguanylate cyclases (DGCs) and c‐di‐GMP‐specific phosphodiesterases (PDEs), which catalyze c‐di‐GMP formation or degradation, respectively (Römling, Galperin, & Gomelsky, 2013; Tal et al., 1998). It has been later shown that DGC activity is associated with the presence of the GGDEF domain, whereas PDEs contain the EAL domain (Ausmees et al., 2001; Simm, Morr, Kader, Nimtz, & Römling, 2004).

Although AAB are known for their ability to oxidize ethanol, tolerance to extremely acidic conditions, and production of cellulose, these features appear to be transient, as they are often rapidly lost when cells are cultured in media without the selective pressure of acetate or ethanol (Beppu, 1993; Coucheron, 1991; Krystynowicz et al., 2002, 2005; Sokollek, Hertel, & Hammes, 1998; Takemura, Horinouchi, & Beppu, 1991). This phenotypic instability has a negative impact on the industrial performance of these species. Studies in various, mainly pathogenic bacteria, have shown that the genetic basis of phenotypic instability has been most commonly caused by spontaneous mutations and genome rearrangements, often associated with genetic elements, such as insertion sequences (IS), genomic islands (GIs), transposable elements (TEs), and transposable bacteriophages (Brzuszkiewicz, Gottschalk, Ron, Hacker, & Dobrindt, 2009; Chan et al., 2015; Kung, Ozer, & Hauser, 2010). Some IS have been found in the genomes of *Komagataeibacter* species, which were associated with cessation of the EPS synthesis (Coucheron, 1993; Iversen, Standal, Pedersen, & Coucheron, 1994; Standal et al., 1994). Moreover, alterations of the plasmid profile have been observed in cellulose‐negative (Cel^−^) cells (Coucheron, 1991). Other genetic elements of the mobile genome of the *Komagataeibacter* genus, however, have not been studied.

Overall, the molecular biology of *Komagataeibacter* has been tested to a limited extend so far and focused mainly on the metabolic pathway of cellulose production from glucose. Furthermore, different laboratories have been using various strains and typically considered only single genes in their analysis (Kubiak, Jedrzejczak‐Krzepkowska, Ludwicka, & Bielecki, 2016). In parallel, many changes in the taxonomic classification bring additional challenges in interpreting the available data. Published results appeared to be very strain‐specific thus making any conclusions about metabolism or regulatory mechanisms, responsible for the BNC production rate, unattainable at the genus level. Therefore, there is a need to broaden the genetic knowledge and to find common features of these strains that drive them to cellulose overproduction. By using the next‐generation sequencing (NGS) technology, we sequenced and assembled the genomes of three strains producing bionanocellulose. These are *K. xylinus* E26 from own collection, *K. xylinus* BCRC 12334, and *K. hansenii* ATCC 53582. Strain *K. xylinus* BCRC 12334 has been exploited mainly in Taiwan (Kuo, Chen, Liou, & Lee, 2016; Wu & Liu, 2012). The strain ATCC 53582, also known as NQ5, has been commonly used in the research worldwide, which can be emphasized by the fact that it has been sequenced by two independent teams during the time of our work (Florea, Reeve, Abbott, Freemont, & Ellis, 2016; Pfeffer, Mehta, & Brown, 2016b). Especially recently, we can observe a steep increase in the number of sequenced *Komagataeibacter* genomes (Pfeffer, Mehta, & Brown, 2016a; Pfeffer, Santos, Ebels, Bordbar, & Brown, 2017a,b,c; Wang et al., 2017; Zhang, Poehlein, Hollensteiner, & Daniel, 2018; Zhang et al., 2017). The growing number of genomic sequences of *Komagataeibacter* strains encouraged us to perform a comparative study with the aim to infer the precise phylogeny of the genus and to investigate sequence conservation in both, the core and the flexible part of the genome. By further focusing on functional diversity among the strains in carbohydrate uptake, EPS biosynthesis, and c‐di‐GMP signaling, we harness the gathered knowledge with the aim to explain the observed phenotypic variability of the in‐house strains.

## 2 MATERIAL AND METHODS

### 2.1 Bacterial strains and growth conditions

Cellulose‐producing strains (*K. xylinus* E26 (from the in‐house collection), *K. xylinus* BCRC 12334 (kind courtesy of Prof. Jyh Ming Wu, Department of Chemical and Materials Engineering, Chinese Culture University, Taipei, Taiwan), and *K. hansenii* ATCC 53582 American Type Culture Collection) were cultured in 10‐ml test tubes filled with 5‐ml of media at 30°C under static conditions for 3 days. One liter culture medium (Hestrin‐Schramm, HS; Hestrin & Schramm, 1954) contained 20.0 g glucose (POCh, Poland), 5.0 g yeast extract (BTL, Poland), 5.0 g bacterial peptone (BTL, Poland), 2.7 g sodium phosphate dibasic (Chempur, Poland), 1.15 g citric acid (Chempur, Poland), and 0.5 g magnesium sulfate (Chempur, Poland). The initial pH of the medium was adjusted to 5.7 with 80% acetic acid (Chempur, Poland). 1% cellulase (from *Trichodrma reesei* ATCC 26921, Sigma‐Aldrich) was added to the culture, and the released cells were harvested for genomic DNA purification.

### 2.2 Genomic DNA isolation and genome sequencing

Genomic DNA from the three strains was isolated according to the procedure published elsewhere (Ausubel et al., 1992) with modifications. Bacterial cells from 3.0 ml of liquid culture were pelleted and washed twice with TGE buffer (25 mM Tris‐Cl; 10 mM EDTA, 50 mM glucose; pH 8) prior to lysozyme (1 mg/ml in TGE) treatment (RT, 30 min). Next, SDS was added to the cells suspension (up to final conc. 0.5%), which was next treated with proteinase K (0.1 mg/ml final conc., Qiagen), at 56°C for 10 min. In the next step, incubation at 65°C for 30 min with CTAB and NaCl (final conc. 1% and 0.7 M, respectively) was applied. After cooling down, the nucleic acids were extracted twice with equal volume of phenol‐chloroform‐isoamyl alcohol (25:24:1) mixture and once with equal volume of chloroform/isoamyl alcohol (24:1) mixture. Finally, RNaseA (Qiagen) hydrolysis was done at 37°C for 20 min followed by additional extraction steps (same as above). Purified genomic DNA was precipitated with 4 M ammonium acetate and 100% ethanol at RT. The DNA pellet was washed twice with 70% ethanol, air‐dried, and suspended in TE buffer. All reagents were from Sigma‐Aldrich if not otherwise stated. NGS libraries were prepared using Nextera XT DNA Library Preparation Kit (Illumina). Genome sequencing was performed using the Illumina MiSeq platform, in 2 × 150 bp paired‐end reads mode. The genomes were sequenced at 20× coverage, on average.

### 2.3 Measurement of carbon source effect on the BNC yield

A pre‐culture was prepared in 5 ml of HS medium (as described above) from one isolated colony and incubated for 72 hr. The final culture was prepared, using 5% inoculum, in 6‐well plates with 10 ml of HS medium in which carbon source (glucose) was replaced with, either fructose, maltose, sucrose, or glycerol. The culture was incubated for 7 days at 30°C. BNC membranes were next treated with 2% solution of NaOH for one night and 1.5% of acetic acid for 4 hr, and then carefully washed in distilled water until neutral pH was reached. The purified membranes were pressed between two filter papers and dried at 80°C in a Gel Dryer apparatus (model 543, Bio‐Rad) until a constant weight was reached (Tiboni et al., 2012). For each strain (*K. xylinus* E25, *K. xylinus* E26, *K. xylinus* BCRC 12334, and *K. hansenii* ATCC 53582 strains) and each carbon source, six cultures (replicates) were prepared.

### 2.4 Scanning electron microscopy

Cellulose biofilms were harvested after 7 days of incubation in 250‐ml flasks, purified by several washes in water and 0.1% NaOH. Finally, water was exchanged into iso‐propanol. Such membranes were deep‐frozen in liquid nitrogen and then freeze‐dried. Before imaging, probes were coated with gold. Membranes’ surfaces were analyzed by Scanning Electron Microscope FEI, Quanta FEG 250, at 40,000× magnification, at Bionanopark sp. z o.o., Lodz, Poland.

### 2.5 Genome assembling and annotation

The sequencing reads were assembled *de novo* using SPAdes (with activated mismatch careful mode and otherwise default settings; v. 3.5.0; Nurk et al., 2013). These Whole Genome shotgun projects were deposited at DDBJ/EMBL/GenBank under the BioProject accession: PRJNA339514 (*K. xylinus* E26), PRJNA339679 (*K. xylinus* BCRC 12334), PRJNA339678 (*K. hansenii* ATCC 53582). The assemblies of the remaining seventeen genomes were downloaded from NCBI (https://www.ncbi.nlm.nih.gov/. (Accessed January 2016); Table 1). Genome statistics for the entire set of twenty genomes were calculated using QUAST (v. 2.3; Gurevich, Saveliev, Vyahhi, & Tesler, 2013). MUM index (MUMi) between pairs of whole genome sequences was generated using Parsnp program with default cutoff settings for maximum MUMi distance (v. 1.2; Treangen, Ondov, Koren, & Phillippy, 2014).

**Table 1:** Properties of the analyzed genomes

Strain	Assembly status	Contigs	N50	Total length [Mbp]	GC%	CDSs	tRNA	tmRNA	rRNA	Cellulose synthesis?	Reference	NCBI assembly ID
***K. xylinus*** **E26**	Contig	366	20387	3.48	62.48	3253	40	1	3	+	TW	BioProject accession: PRJNA339514
***K. xylinus*** **BCRC 12334**	Contig	306	29323	3.66	62.43	3386	40	1	3	+	TW	BioProject accession: PRJNA339679
***K. hansenii*** **ATCC 53582**	Contig	252	49082	3.39	59.42	2952	45	1	3	+	TW	BioProject accession: PRJNA339678
*Ga. diazotrophicus* PAl 5	Complete Genome	3	NA	4.00	66.33	3740	60	1	12	−	Bertalan et al. (2009), Valera, Torija, Mas, and Mateo (2015) and Valera, Poehlein, et al. (2015)	ASM6704v1
*K. europaeus* 5P3	Scaffold	256	39631	3.99	61.49	3773	54	2	15	ND	Andrés‐Barrao et al. (2011)	ASM28533v1
*K. europaeus* CECT 8546	Contig	116	103383	4.11	61.31	3869	55	1	9	+	Valera, Torija, Mas, and Mateo (2015) and Valera, Poehlein, et al. (2015)	ASM127364v1
*K. europaeus* LMG 18494	Scaffold	216	64353	3.99	61.24	3738	59	1	24	ND	Andrés‐Barrao et al. (2011)	ASM22754v1
*K. europaeus* LMG 18890^T^	Scaffold	321	35510	4.23	61.26	4050	51	2	12	−	Andrés‐Barrao et al. (2011), Valera, Torija, Mas, and Mateo (2015) and Valera, Poehlein, et al. (2015)	ASM28529v1
*K. europaeus* NBRC 3261	Scaffold	596	27771	3.63	61.6	3460	41	2	3	ND	NP	ASM96448v1
*K. hansenii* ATCC 23769	Chromosome	1	NA	3.64	59.5	3186	45	1	1	+	Iyer, Geib, Catchmark, Kao, and Tien (2010), Valera, Torija, Mas, and Mateo (2015) and Valera, Poehlein, et al. (2015)	ASM16439v1
*K. hansenii* JCM 7643	Scaffold	467	25111	3.71	59.29	3275	40	1	3	+	Valera, Torija, Mas, and Mateo (2015) and Valera, Poehlein, et al. (2015)	ASM96440v1
*K. intermedius* AF2	Scaffold	377	59913	4.52	61.34	4222	45	1	12	+	Dos Santos et al. (2015)	ASM81725v1
*K. intermedius* TF2	Contig	943	61064	3.88	61.67	3511	39	1	3	ND	NP	ASM96442v1
*K. kakiaceti* JCM 25156	Contig	947	5713	3.13	62.14	3859	35	1	3	+	Iino et al. (2012)	ASM61330v1
*K. medellinensis* NBRC 3288	Complete Genome	8	NA	3.51	60.58	3348	57	1	15	+/−	Matsutani et al. (2015) and Ogino et al. (2011)	ASM18274v1
*K. oboediens* 174Bp2	Scaffold	200	74982	4.18	61.26	3996	66	0	30	+	Andrés‐Barrao et al. (2011)	ASM22756v1
*K. rhaeticus* AF1	Scaffold	213	73183	3.94	62.49	3647	53	1	5	+	Dos Santos et al. (2014)	GLUCORHAEAF1_v1
*K. sp*. SXCC‐1	Contig	64	162897	4.23	62.44	3908	59	1	15	ND	Du, Jia, Yang, and Wang (2011)	ASM20863v1
*K. xylinus* E25	Complete Genome	6	NA	3.91	62.13	3671	59	1	15	+	Kubiak et al. (2014)	ASM55076v1
*K. xylinus* NBRC 13693	Contig	211	70122	3.34	61.95	3024	46	1	3	+	NP	ASM96450v1

Note

In bold are given genomes, which were sequenced in this work. Total assembly length for each genome was calculated by adding all genome sequences (chromosomes, plasmids, contigs, or scaffolds). Total number of predicted genes coding for rRNA (5S/16S/23S) is given in the “rRNA” column. +/−: the strain synthesizes/does not synthesize cellulose; NA: not applicable; ND: not determined; NP: no publication; TW: this work.

To check the presence of plasmid *repA* gene in the sequenced draft genomes, we searched them with the sequences of plasmid RepA protein (based on NCBI annotation) from the plasmids of the completely sequenced *Komagataeibacter* genomes (*K. europaeus* SRCM101446, pKE1446‐1 (WP_087609090.1); *K. xylinus* E25, pGX3 (WP_081749530.1); *K. medellinensis* NBRC 3288, pGXY020 (WP_007284615.1); *K. nataicola* RZS01, pKNA02 (WP_078528475.1), pKNA03 (WP_078528546.1), pKNA04 (WP_078528626.1)) by using tblastn program and applying a hit validity threshold of 50% identity and 70% coverage.

Genome sequences were next annotated using Prokka (default settings; v. 1.11; Seemann, 2014) employing: Prodigal (protein‐coding gene prediction; v. 2.6; Hyatt et al., 2010); Aragorn (transfer RNA gene prediction; v. 1.2; Laslett & Canback, 2004); Barrnap (ribosomal RNA gene prediction; v. 0.7; http://www.vicbioinformatics.com/software.barrnap.shtml); Infernal (noncoding RNA prediction; v. 1.1.2; Kolbe & Eddy, 2011). Proteins of *Komagataeibacter* strains were additionally annotated using software package InterProScan (v. 5.19; Jones et al., 2014) with option to scan for Pfam collection of protein families. A close inspection was given to the proteins, which had only one of the GGDEF/EAL (Pfam: PF00563/PF00990) domains. The single‐domain proteins were mostly those, which harbored a short EAL domain and lay at the start of a contig. It was verified with *K. xylinus* E26 and *K. xylinus* BCRC 12334 assemblies that for those genes, the proceeding contig matched GGDEF domain sequence, however, was not predicted by Prokka, since it lacked a stop codon, due to contig truncation, which is a commonly observed drawback of draft genomes.

### 2.6 Orthologous proteins analysis and core genome calling

The clusters of orthologous proteins in the analyzed genomes were generated using Proteinortho program (v. 5.11; Lechner et al., 2011). When it was necessary, gene presence was verified using the NCBI BLAST program (version 2.2.26; Altschul, Gish, Miller, Myers, & Lipman, 1990; Camacho et al., 2009). The core genome set was built by finding clusters of genes, which had an ortholog in every *Komagataeibacter* genome (*G. diazotrophicus* PAl 5 was not included). Genomes of *K. kakiaceti* JCM 25156 and *K. intermedius* TF2, were excluded from this list due to low genome quality. Hierarchical clustering and heatmap plotting was done using R (v. 3.1.0) and gplots package (release 2.17.0). CLC Sequence Viewer (v. 7.8.1) was used to generate and visualize multiple sequence alignments. SnapGene Viewer (v. 3.3.4) was used to display structure of gene clusters.

### 2.7 Phylogenetic analysis

The predicted 16S rRNA sequences (DNA) were aligned using MUSCLE (Edgar, 2004) and the phylogenetic analysis was conducted using MEGA (v. 6.06; Tamura, Stecher, Peterson, Filipski, & Kumar, 2013). Phylogenetic tree was constructed using the Maximum Likelihood method and by employing the Hasegawa‐Kishino‐Yano DNA substitution model with GAMMA distributed rate variation among sites model (HKY+G). The reliability of the tree was estimated using bootstrap method with 500 replicates. For the phylogenetic tree based on the orthologous protein sequences, the original orthologs list was filtered for single copy genes present in all twenty genomes, which resulted in 868 clusters. The sequences of these proteins were aligned in their ortholog sets using MAFFT (v. 7.305; Katoh, Misawa, Kuma, & Miyata, 2002). These alignments were next concatenated. The Maximum Likelihood tree was searched using RAxML (v. 8.2.69; Stamatakis, 2014) under the GAMMA model of rate heterogeneity and with automatic protein substitution model selection. Number of bootstrap replicates was selected using automatic bootstrapping criteria, as implemented in the program. The trees generated using RAxML were drawn in FigTree (v. 1.4.3; http://tree.bio.ed.ac.uk/software/figtree/).

### 2.8 Genomic islands, insertion sequences, prophages, and CRISPR‐Cas loci prediction

Genomic islands were predicted using IslandViewer3 online service (http://www.pathogenomics.sfu.ca/islandviewer/. (Accessed March 2016); Dhillon et al., 2015). Insertion sequences were predicted using ISsaga online service on the chromosome sequences of the complete genomes and of *K. hansenii* ATCC 23769 strain (http://issaga.biotoul.fr/issaga_index.php. (Accessed March 2016); Varani, Siguier, Gourbeyre, Charneau, & Chandler, 2011). Prophage prediction was done using PHASTER online service on unannotated chromosome sequences of the complete genomes and the chromosome sequence of *K. hansenii* ATCC 23769 strain (NZ_CM000920.1 for *K. hansenii* ATCC 23769; NC_010125.1 for *G. diazotrophicus* PAl 5; NZ_CP004360.1 for *K. xylinus* E25; NC_016027.1 for *K. medellinensis* NBRC 3288 (http://phaster.ca/ (Accessed November 2017); Arndt et al., 2016). Clustered Regularly Interspaced Short Palindromic Repeats (CRISPRs) were predicted in all analyzed genomes using MinCED program (v. 0.2.0; https://github.com/ctSkennerton/minced). Additionally, predictions were made using CRISPRFinder web tool (http://crispr.i2bc.paris-saclay.fr/Server/ (Accessed March 2016); Grissa, Vergnaud, & Pourcel, 2007). Only the confirmed loci were considered. The results generated by both programs agreed in the majority of cases.

### 2.9 Functional enrichment of the core genome

The proteome of *K. xylinus* E25 was annotated using COG through WebMGA server of Wei Zhong Li Lab (http://weizhongli-lab.org/metagenomic-analysis/server/cog/ (Accessed April 2016). In total, 2,461 genes were assigned to at least one COG category, which was the case for 1,402 proteins of the core genome set. Additionally, this proteome was annotated using RAST annotation service (Aziz et al., 2008). In this case, 1,350 genes were annotated with at least one RAST category. For every COG or RAST category, assigned proteins were counted in the entire and in the core genome. Multi‐category proteins (assigned to more than one COG/RAST category) were counted in each of their categories. Functional enrichment was conducted using Fisher's exact test (one‐tailed) as implemented in R. FDR was controlled by using Benjamini & Hochberg method (1995).

## 3 RESULTS AND DISCUSSION

### 3.1 Phenotypic and genomic diversity of the *Komagataeibacter* strains

**Figure 1:**
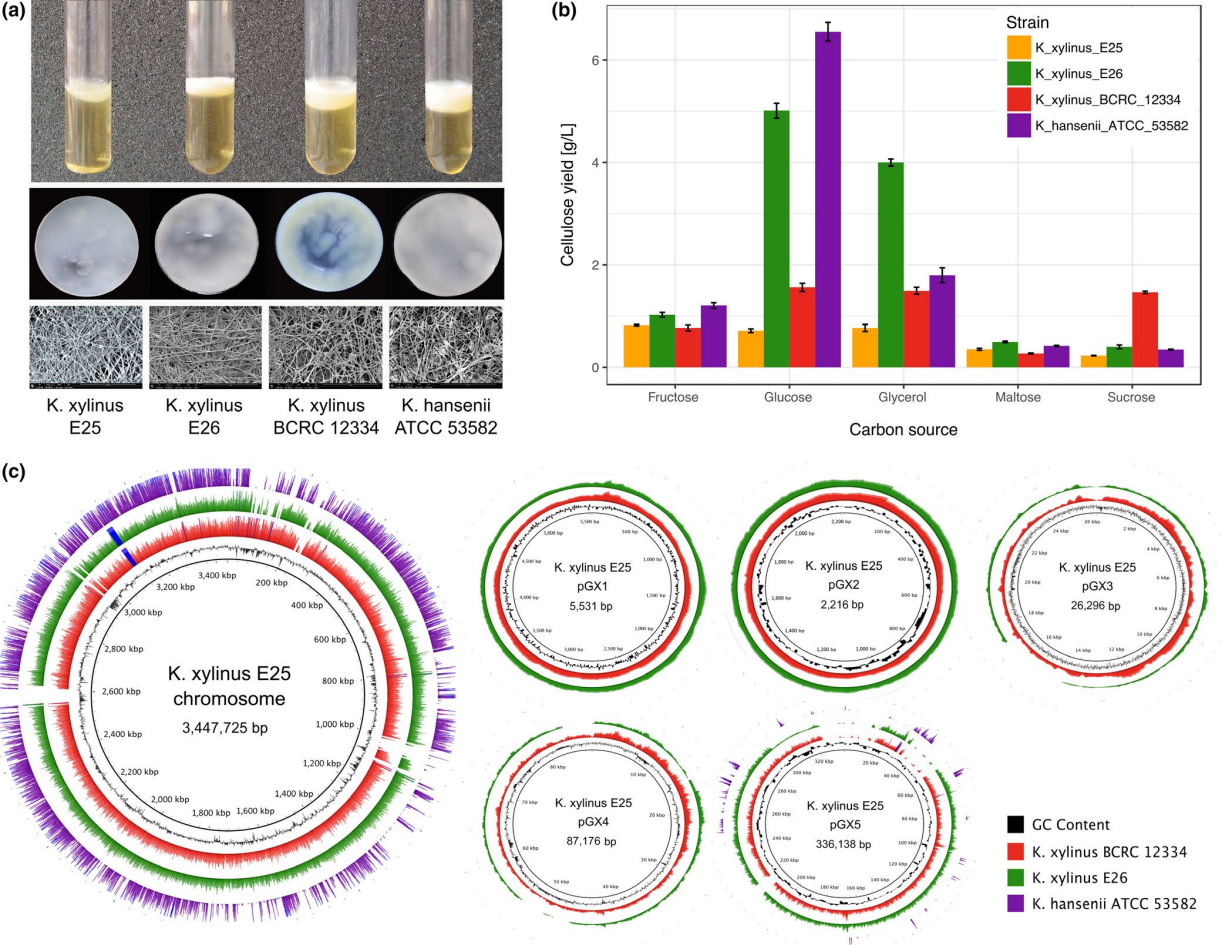
Phenotypic and genomic characteristics of the in‐house strains. (a) Comparison of cellulose pellicles. Liquid culture (top panel), macroscopic (middle panel), and SEM pictures (bottom panel) of membranes. (b) Cellulose yield in media containing five different carbon sources (fructose, glucose, glycerol, maltose, and sucrose). Error bar represents standard deviation calculated from six replicated cultures. (c) Mapping coverage of sequencing reads from three NGS libraries (color rings: *K. xylinus* E26—green, *K. xylinus* BCRC 12334—red, *K. hansenii* ATCC 53582—purple) to the genome of *K. xylinus* E25 (chromosome and five plasmids)

We have observed that four *Komagataeibacter* strains from the in‐house collection displayed phenotypic differences related to the produced cellulose pellicle (Figure 1a). Each of pellicles differed in microscopic arrangements of fibrils, which formed pores of various sizes. Consistent with previous reports, one of the strains, *K. hansenii* ATCC 53582, produced wider cellulose fibers (Czaja et al., 2007). Furthermore, the four strains from the in‐house collection varied in cellulose productivity when grown on different carbon sources with glucose and glycerol being most preferable, whereas maltose was the least preferable one (Figure 1b). Similar discrepancies have been published in independent studies testing other cellulose‐producing strains (Keshk & Sameshima, 2005; Mikkelsen, Flanagan, Dykes, & Gidley, 2009; Nguyen, Flanagan, Gidley, & Dykes, 2008). The most explicit differences between all tested strains were pronounced when grown on glucose. Specifically, *K. hansenii* ATCC 53582 and *K. xylinus* E26 strains productivity was four and three times higher as compared to the other two *K. xylinus* strains, respectively. Interestingly, we noticed exceptionally high cellulose productivity for the *K. xylinus* E26 strain grown on the medium with glycerol as the main carbon source and *K. xylinus* BCRC 12334 strain grown on sucrose (Figure 1b). Another important difference between the phenotypes of the investigated strains is stability of cellulose production. Among the four in‐house strains compared here, *K. hansenii* ATCC 53582 strain was the most productive in submerged cultures (data not shown).

To understand the genetic basis of the observed phenotypic differences between the three *K. xylinus* in‐house strains and the *K. hansenii* ATCC 53582 strain, their genomes were sequenced using the NGS technology. Since the complete genome of *K. xylinus* E25 strain was already available (Kubiak et al., 2014), the remaining three genomes were sequenced. The sequencing was conducted in a cost‐effective manner, at low coverage of approximately 20×. The assembled draft genomes consist of 300 contigs, on average (Table 1). The assembly of *K. hansenii* ATCC 53582 genome is of the highest quality (the highest contiguity) and has the smallest GC content among the sequenced genomes (Table 1). Moreover, mapped read sequences of *K. xylinus* E26 and *K. xylinus* BCRC 12334 strains, unlike those of *K. hansenii* ATCC 53582 strain, covered the genome of *K. xylinus* E25 strain almost completely (Figure 1c).

Next, we estimated genomic distances between these four genomes using the MUM index, which is based on the number of maximal unique and exact matches shared by two genomes and takes values between 0 and 1, for very similar and very diverged genomes, respectively (Deloger, El Karoui, & Petit, 2009). This analysis confirmed that the genomes of *K. xylinus* E25, *K. xylinus* E26, and *K. xylinus* BCRC 12334 strains are much more similar to one another (MUMi values close to 0), whereas, for *K. hansenii* ATCC 53582 strain, MUMi values were of approximately 0.5, suggesting higher sequence divergence with the other three strains. Further comparative genome analysis produced a list of SNPs, InDels and missing or unique genes of mostly unknown function (data not shown). Finding which of these differences affect cellulose phenotype would be challenging without carrying out more experimental studies. What is more, other genomic differences, such as genome rearrangements, cannot be accurately investigated at the draft level of genome assembly. Therefore, we decided to include more of *Komagataeibacter* genomes and to perform a wide comparative study to extract consistent genomic patterns characterizing the genus and highlighting the most meaningful differences.

**Figure 2:**
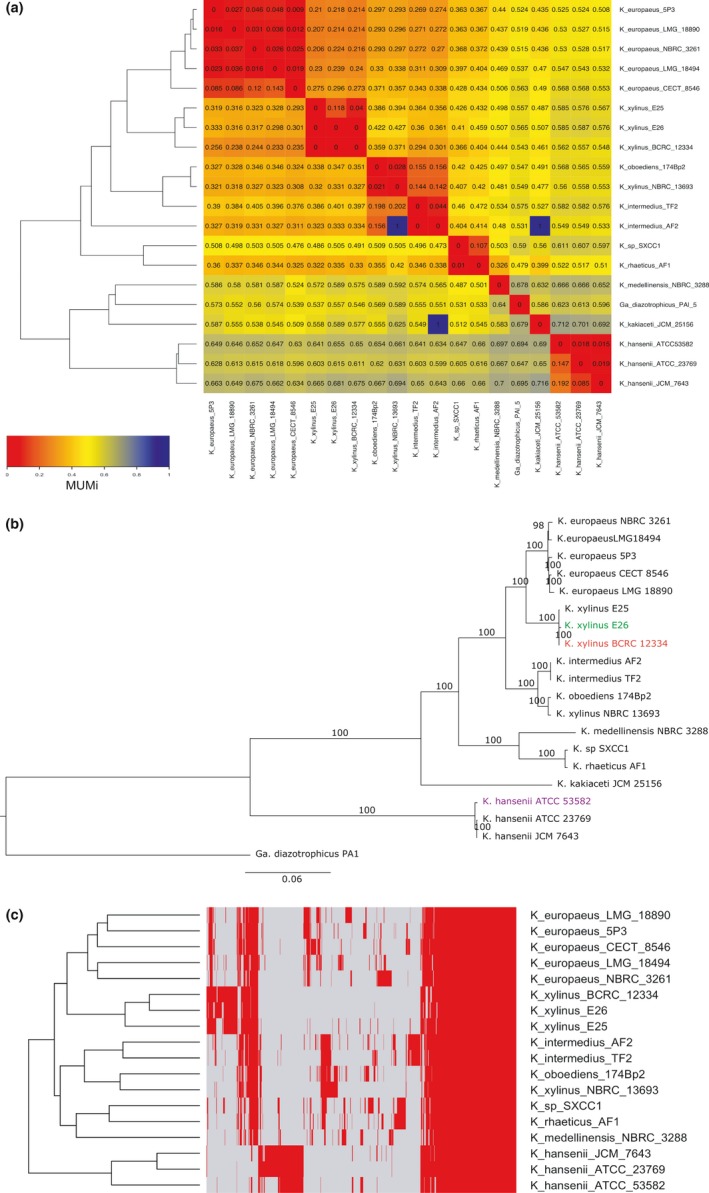
Relationship between *Komagataeibacter* genomes. (a) Reciprocal MUMi values between each of the 20 analyzed *Komagataeibacter* and a *Gluconacetobacter diazotrophicus* PAl 5 genome. Color scale from red to blue corresponds to lowest (highest similarity) and highest (lowest similarity) MUMi values, respectively. Dendrogram was generated based on hierarchical clustering analysis. (b) Maximum Likelihood phylogenetic tree calculated based on sequences of 868 orthologous proteins. Colored are the newly sequenced strains of *K. xylinus* E26 (green), *K. xylinus* BCRC 12334 (red), and *K. hansenii* ATCC 53582 (purple). The bar represents 6% sequence divergence. The numbers above branches represent bootstrap values. The tree was generated in RAxML and drawn in FigTree. (c) Clustering of *Komagataeibacter* strains based on presence (red)/absence (gray) of orthologs pattern. Dendrogram was generated based on hierarchical clustering analysis. *Y*‐axis: strains clustering; *x*‐axis: protein clustering (dendrogram not shown). *K. kakiaceti* JCM 25156 is not included due to poor quality of its genome

We annotated *de novo* our and publicly available genome sequences of *Komagataeibacter* strains using Prokka (Seemann, 2014). Additionally, one complete genome of *Gluconacetobacter diazotrophicus* PAl 5*,* a well‐studied free‐living strain, was included as a reference. Table 1 presents the general characteristics of the twenty analyzed genomes. Only three genomes of this set are completely assembled, whereas the others are in a draft state, with a varying degree of quality. The genome length of *Komagataeibacter* strains is in the range of 3.13–4.52 Mbp. The DNA G+C content is rather similar and in the range of 59%–62%, with the *K. hansenii* strains displaying the lowest values (Supporting Information Figure S1). Next, we generated a cladogram based on the MUM index to gain a rough overview of the sequence similarity among the twenty genome sequences. This analysis grouped genomes into several clades according to reciprocally low values (0–0.2; Figure 2a). Three main clades can be distinguished based on this cladogram. One clade groups the *K*. *europaeus,* the *K. xylinus, K*. *oboediens*, the *K. intermedius, and K. rhaeticus* strains. In general, the *K*. *hansenii* strains scored much higher MUMi values with other genomes suggesting higher sequence divergence. On the other hand, *K. medellinensis* NBRC 3288 and *K. kakiaceti* JCM 25156 genomes localize in between of the *K*. *xylinus* and the *K*. *hansenii* clades.

### 3.2 Phylogenetic analysis and the core genome of the *Komagataeibacter* genus

In order to verify the cladding pattern based on MUMi values, we performed the phylogenetic analysis based on the 16S rRNA sequence. This yielded a tree of low confidence, as tested by bootstrap test (Supporting Information Figure S2A). Moreover, on this tree, many of the *K*. *xylinus* strains clustered together with the *K*. *europaeus* strains. To perform a more accurate phylogenetic classification, we searched for a stronger taxonomic signal than a single gene sequence. We decided to use all available genomic data. For this purpose, orthologs were searched among their proteomes, which resulted in generation of 6,724 clusters holding proteins from at least two strains. Out of these, 1,578 orthologous gene clusters are present in every *Komagataeibacter* genome (constituting the core genome). After further filtering of the core genome set to select those clusters, which hold only single copy genes, we chose a set of 868 ortholog clusters. The phylogenetic analysis based on this group yielded a tree separating the genomes into species‐specific clades (Figure 2b). The tree topology was further confirmed with a previously proposed method, which employed *dnaK‐groEL‐rpoB* genes only (Supporting Information Figure S2B; Cleenwerck, De Vos, & De Vuyst, 2010). The advantage of using genomic data is manifested by a higher reliability, as measured by the bootstrap test. At this stage, it is clear that two distinct clades divide the genus, one that groups the *K*. *europaeus*, the *K. xylinus*, the *K*. *intermedius*,* K*. *oboediens* 174Bp2, *K*. *medellinensis* NBRC 3288, *K*. *rhaeticus* AF1, and *K*. *kakiaceti* JCM 25156 strains (from now on referred to as the *K. xylinus* clade in this work); the second clade constitutes of the three *K*. *hansenii* strains (now on called in this work the *K. hansenii* clade).

Additionally, we tested the sharing pattern of the entire orthologs set (Supporting Information Figure S3). Here, again the fewest orthologs were shared between the *K. hansenii* and the *K. xylinus* clade. Next, we focused on the groups of orthologs, which are not shared by all *Komagataeibacter* strains by investigating the cladding of their presence/absence pattern (Figure 2c). Roughly three major orthologs’ clusters were formed, one, the biggest, grouping genes present in every *Komagataeibacter* genome (genes clustered at the right side of the Figure 2c); one characteristic of the *K*. *xylinus* species; and another one distinguishing the *K. hansenii* strains. In general, the distinction of the *K. hansenii* strains from the rest of the *Komagataeibacter* spp. presented here is in accordance with previous studies (Andrés‐Barrao et al., 2013; Cleenwerck et al., 2010).

Based on our results, the two strains sequenced in this work, *K. xylinus* E26 and *K*. *xylinus* BCRC 12334, clustered together with *K. xylinus* E25, whereas the reference strain *K. hansenii* ATCC 53582 clearly grouped with other *K. hansenii* strains. The other *K. xylinus* strain, NBRC 13693, clustered rather far from *K. xylinus* E25, which was unexpected. To clarify these taxonomy differences, we decided to include the partial sequences for *dnaK*,* groEL*, and *rpoB* genes of the type strains from the work of Cleenwerck et al. (2010). The resulting tree showed that the majority of the strains are correctly classified at the species level (Supporting Information Figure S4). However, the *K. xylinus* genomes, contributed by our work (*K. xylinus* E25, *K. xylinus* E26, *K. xylinus* BCRC 12334) as well as *K. xylinus* NBRC 13693, cluster far from *K. xylinus* LMG 1515, the *K. xylinus* type strain (Yamada, 2016). Although, the *K. xylinus* E25, *K. xylinus* E26, and *K. xylinus* BCRC 12334 strains position close to the *K. swingsii* LMG 22125 (isolated from apple juice in South Tyrol region in Italy), they form a distinctive clade in this tree. These results suggest that *K. xylinus* NBRC 13693 probably belongs to *K. oboediens*;* K. sp*. SXCC1 is likely a *K. rhaeticus* strain, whereas the *K. xylinus* strains contributed by this work formed a group, which could be separated as species or subspecies, when more data would accumulate.

### 3.3 The mobile genome elements in *Komagataeibacter* strains

Sequence‐based and phylogenetic analysis conducted so far defined the core genome of the tested *Komagataeibacter* strains and enabled their classification, suggesting the clear separateness of the *K. hansenii* clade. Next, we focused on the mobile part of *Komagataeibacter* genomes (namely plasmid DNA, genomic islands GIs, insertion sequences IS, and prophages) because phenotypic diversity among bacterial strains of the same species is typically connected with this portion of DNA (Darmon & Leach, 2014).

#### 3.3.1 Plasmids

The diversity among plasmid DNA has already been connected with phenotypic changes for other AAB strains (Akasaka et al., 2015; Azuma et al., 2009). Conjugative plasmid DNA transfer, in cellulose‐producing and cellulose nonproducing derivatives of *K. xylinus* ATCC 10245 (in the original paper: *Acetobacter xylinum* ATCC 10245) strain, has been shown in pioneering studies of Jackson, Vinatzer, Arnold, Dorus, and Murillo (2011). The complete genomic sequences of *Komagataeibacter* strains (*K. xylinus* E25 (Kubiak et al., 2014), *K. medellinensis* NBRC 3288 (Ogino et al., 2011), and the two strains deposited during the time of preparation of this manuscript—*K. nataicola* RZS01—(Zhang et al., 2017) and *K. europaeus* SRCM 101446; unpublished) have shown the presence of five, seven, six, and three plasmids in these strains, accordingly. In case of the three in‐house strains sequenced here, the results of mapping of sequencing reads to the *K. xylinus* E25 genome suggested the presence of at least two small plasmids (~5 kbp and ~2 kbp) in *K. xylinus* E26 and *K. xylinus* BCRC 12334 strains (Figure 1c). However, the depth of sequencing was not sufficient for proper plasmid sequence reconstruction, and therefore, we verified this prediction by identification of plasmid replication protein RepA in the whole‐genome assemblies. RepA homologs were detected on two separate contigs in *K. xylinus* E26 and *K. xylinus* BCRC 12334 genomes and one on one contig of *K. hansenii* ATCC 53582 genome (Supporting Information Table S1). Together with the published results, our data suggest that the variability in plasmid DNA content may contribute to the phenotypic diversity of the *Komagataeibacter* strains.

#### 3.3.2 Short mobile genetic elements prediction

Two best‐studied groups of short mobile elements commonly related to bacterial strains phenotypic diversity are insertion sequences (ISs) and prophages. There are well‐established bioinformatics tools enabling prediction of these mobile sequences available. In order to assure accuracy, complete assemblies of genomes are required. Therefore, four chromosome sequences from the completed or closed genomes (Table 1) were used. Results showed a tendency of lower number of both, IS and prophage sequences, in *K. hansenii* ATCC 23769 strain when compared to *K. xylinus* E25 and *K. medellinensis* NBRC 3288 strains (Supporting Information Figure S5). The last‐mentioned strain shows a similar level of short mobile sequences as compared to the free‐living *G. diazotrophicus* PAI 5 strain used as a reference. The only mobile elements reported before in *Komagataeibacter* strains (namely *K. hansenii* ATCC 23770 and *K. hansenii* ATCC 23769) are insertion sequence IS 1031 and its derivatives (Coucheron, 1991, 1993; Standal et al., 1994). In contrast, our predictions showed quite complex composition of the IS families; for example, fifteen of them were identified in *K. xylinus* E25 genome (Supporting Information Figure S6). Absence of prophage sequences is very unusual in bacterial genomes but has been reported previously, for example, for *Acetobacter pasteurianus* 386B strain—a stable and industrially exploited AAB representative (Illeghems, De Vuyst, & Weckx, 2013). On the other hand, the presence of numerous diverse prophages is often linked with short‐term strain variation, for example, in *Streptococcus pneumoniae* lineages (Croucher et al., 2014). Therefore, the relatively low number of insertion sequences and lack of intact prophages in *K. hansenii* ATCC 23769 strain genome may be interpreted as an indication of the highest stability of this genome among the compared three representatives of the *Komagataeibacter* genus.

#### 3.3.3 Genomic islands prediction

**Figure 3:**
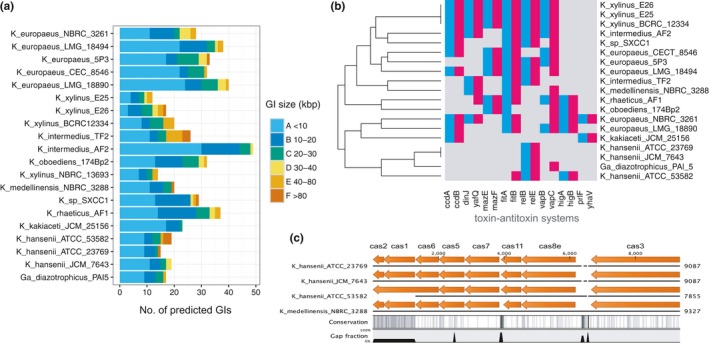
The flexible genome characteristics. (a) Number of predicted GIs of various sizes (kbp) in all analyzed 20 genomes of *Komagataeibacter* and a *Gluconacetobacter diazotrophicus* PAl 5 genome. (b) Presence (colored)/absence (gray) map of toxin–antitoxin systems in *Komagataeibacter* and a *Gluconacetobacter* strains. Antitoxins are marked in blue, toxins in red and arranged consecutively in pairs. Dendrogram (*y*‐axis: strains clustering) was generated based on hierarchical clustering analysis. (c) CRISPR‐Cas gene cluster in *K. hansenii* strains and *K. medellinensis* NBRC 3288 constituting of eight *cas* genes. In *K. hansenii* ATCC 53582, *cas*1 is incomplete, as it is located at a contig border; *cas*2 is present on another contig. Figure produced using SnapGene and modified

In contrast to prophages and ISs, genomic islands (GIs) are linked with more ancient evolutionary events and frequently are composed of genes of horizontal transfer origin, which appeared to be beneficial for a bacterial cell (Croucher et al., 2014; Gyles & Boerlin, 2014). Interestingly, genes encoded on GIs may take part in attenuation of other genetic elements’ mobility (Darmon & Leach, 2014). The occurrence of genomic islands in all nineteen analyzed genomes was predicted using the closest complete (or closed) sequence as reference (the chromosome of *K. hansenii* ATCC 23769 for the *K. hansenii* strains and *K. xylinus* E25 for the remaining draft genomes). It should be stated here, that, inconsistently with Island Viewer's settings, a GI should have length of at least 10 kbp, according to the most frequently used definition (Bellanger, Payot, Leblond‐Bourget, & Guédon, 2014). The pattern of predicted genomic islands (in the meaning of size and number) varied for the two main intragenus clades. The lowest number of GIs was found in the *K. hansenii* strains, whereas the opposite result was obtained in all of the *K. europaeus* strains (Figure 3a). Large number of diverse mobile genetic elements are of the key importance, for example, in the survival in extreme environments for the acidophilic *Acidithiobacillus caldus* ATCC 51756 strain (Acuña et al., 2013); and in maintaining virulence of numerous pathogens, including enterotoxigenic *Escherichia coli*,* Salmonella typhimurium*,* Streptococcus pyogenes,* and *Clostridium perfringens* (Gyles & Boerlin, 2014). It was expected to find the source of phenotypic divergence among the in‐house *K. xylinus* strains in the sequences or orthologs’ content of the identified genomic islands (Supporting Information Figure S7A). It appeared that only a few rearrangements on GI sequences could be found and that in each of the three analyzed strains, only 13‐20% of GI‐localized genes were unique (Supporting Information Figure S7B). Among unique sequences, genes connected with DNA rearrangements, transcriptional regulation, and cellular stress response (e.g., error‐prone polymerases, HTH‐transcriptional regulators, type IV secretion system genes and chaperons) were found, suggesting indirect influence on cellulose production process. There was a huge predominance of CDSs annotated as hypothetical proteins among them (namely: 68% in *K. xylinus* E25, 69% in *K. xylinus* E26, and 80% in *K. xylinus* BCRC 12334 strain), causing difficulty in posing any hypothesis about the functional pathways adopted on these islands.

#### 3.3.4 Predicted mechanisms regulating flexibility of *Komagataeibacter* genomes

Mobile genetic elements are under control of protective molecular mechanisms, like toxin antitoxin systems (TA) found on GIs or CRISPR‐Cas systems, which sustain a balance between genome flexibility and stability assuring cell survival (Darmon & Leach, 2014). First, we tested for the presence of TA systems in the studied genomes, using our orthologous groups identified previously. Only one toxin‐antitoxin system (namely *fit*A/B) was found as common in all *Komagataeibacter* strains tested with the exception of *K. kakiaceti* JCM 25156 and the whole *K. hansenii* clade, in which *rel*B/E system is primarily found (Figure 3b).

Surprisingly, we found that only the *K. hansenii* clade and *K. medellinensis* NBRC 3288 strain harbors a CRISPR‐Cas system, in particular, of the Type I‐E, as predicted by the presence of *cas3* signature gene and sequence similarity to *E. coli* K12 proteins (Figure 3c; Choi & Lee, 2016; Makarova, Zhang, & Koonin, 2017). These genomes contain one loci of the *cas* gene cluster consisting of 4‐20 palindromic repeats of 29 bp, separated by 32‐bp spacers (Supporting Information Table S2). We searched the NCBI nucleotide database with spacer sequences of the *K. hansenii* strains but were unable to find any valid match with a viral, prophage or plasmid sequences. Only one spacer from *K. medellinensis* NBRC 3288 shared a moderate sequence similarity with the pAC58‐29 plasmid from *Acetobacter pasteurianus* AC2‐58 (data not shown). Therefore, speculation about foreign DNA invasion events, “remembered” by CRISPR‐Cas system, is impossible at this stage of knowledge. Higher representation of TA systems in the strains possessing numerous IS and/or prophage sequences (e.g., *K. medellinensis* NBRC 3288 or *K. xylinus* E25 strains) seems reasonable. Moreover, the *K. hansenii* clade again showed to be clearly separated from all the rest of the analyzed strains with the smallest number of toxin–antitoxin systems and unique presence of CRISPR‐Cas system. The importance of the CRISPR‐Cas system in maintaining genome stability in other AAB representatives, *Acetobacter pasteurianus* CICC 20001 and CGMCC 1.41 strains and their derivatives, was proved recently (Wang, Shao, Chen, Chen, & Chen, 2016), but no such studies have been done for the *Komagataeibacter* genus so far.

### 3.4 Functional diversity of the *Komagataeibacter* genus

Ortholog‐conservation pattern displayed distinctive clusters, which may group proteins responsible for species‐specific features (Figure 2c). Investigation of these clusters, however, did not expose any particular functional groups among them, likely due to poor annotation (data not shown). In the next steps, we focused on functional analyses of the core genome. For this purpose, we investigated functions represented by the core genes. To do this, we performed functional enrichment, based on the COG and RAST functional categories, in respect t*o K. xylinus* E25 genome annotation only (Supporting Information Figure S8A,8B). This analysis revealed a few overrepresented housekeeping COG categories, such as nucleotide transport and metabolism (F); coenzyme transport and metabolism (H); translation, ribosomal structure and biogenesis (J); posttranslational modification, protein turnover, chaperones (O). This could suggest some divergence among other functional groups and thus higher than expected intragenus functional diversity of the *Komagataeibacter* strains. Nevertheless, a meaningful analysis is not yet possible for these species, since functional categories, either COG or RAST, were assigned to only ca. 30% of the proteins in *K. xylinus* E25 genome. Therefore, we further concentrated on the most characteristic features, such as carbon source preference and exopolysaccharides production, including cellulose.

#### 3.4.1 Predicted carbohydrate uptake mechanisms

One of the surprising results revealed by functional enrichment was the lack of “Carbohydrates” category (Supporting Information Figure S8A,S8B) since soluble exopolysaccharides and cellulose secretion are regarded as shared features of the genus. In our four in‐house strains, we observed variability in the utilization of the two carbon sources that promote the highest cellulose yield, that is, glucose and glycerol (Figure 1b). Comparative analysis between *K. xylinus* E25, *K. xylinus* E26, and *K. xylinus* BCRC 12334 strains showed a very high sequence conservation of the proteins involved in glucose metabolism and cellulose biosynthesis, such as periplasmic PQQ‐dependent glucose dehydrogenase, glucokinase, glucose‐6‐phosphate dehydrogenase (and its isoforms), phosphoglucomutase, UDP‐glucose pyrophosphorylase, cellulose synthase subunits (BcsA, BcsB, BcsC, BcsD) (Chawla, Bajaj, Survase, & Singhal, 2009; Kuo, Teng, & Lee, 2015) (data not shown). Therefore, it seemed that genetic basis contributing to the differences in cellulose yield in these strains is likely not related to the direct catalytic machinery. For this reason, we decided to further investigate carbohydrate uptake and exopolysaccharides production pathways. Although a variety of carbohydrate transport systems have been characterized in bacteria, none has been experimentally investigated in this genus. The availability of *Komagataeibacter* genomes allows now making predictions based on sequence similarity to known channels and transporters.

#### 3.4.2 Predicted glucose uptake mechanism

**Figure 4:**
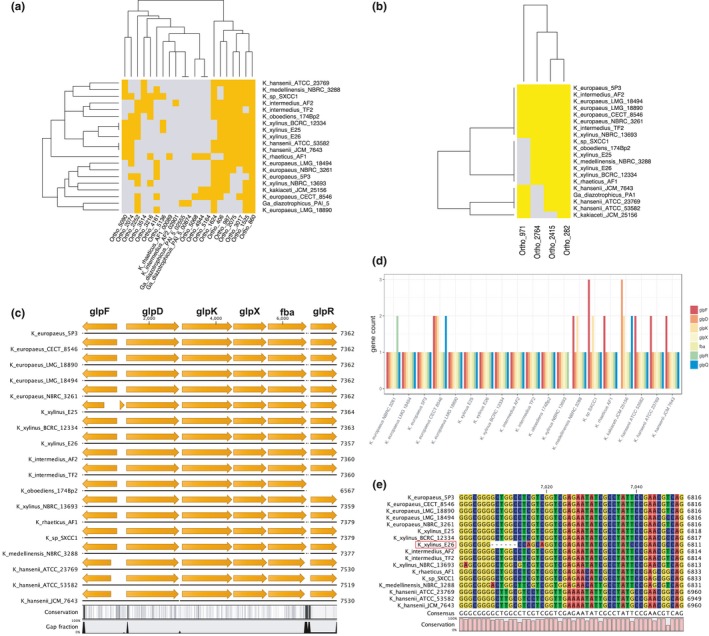
Conservation of glucose and glycerol transporters, and metabolic enzymes. (a) Presence (gold)/absence (gray) map of OprB proteins. (b) Presence (yellow)/absence (gray) map of GalP proteins. In both panels, (a and b), dendrograms were generated based on hierarchical clustering analysis. *Y*‐axis: strains clustering; *x*‐axis: protein clustering. (c) Conservation of glycerol metabolism operon. Aligned are only operon sequences contained within intact contigs. The gene symbols relate to putative functions: *glpF*—membrane diffusion facilitator for glycerol; *glpD*—aerobic G3P dehydrogenase; *glpK*—cytoplasmic glycerol kinase; *glpX*—fructose‐1,6‐bisphosphatase; *fba*—fructose‐bisphosphate aldolase*; glpR*—repressor of the *glp* regulon. (d) Number of *glp* genes in *Komagataeibacter* genomes. (e) Multiple sequence alignment of GlpR protein. Panels c and e generated using CLC Sequence Viewer

In gram‐negative bacteria, glucose must pass through two membranes before it enters the cytosol. The permeability of the outer membrane is often dependent on the channels formed by porins. Based on Prokka annotation and InterProScan search, one candidate for a glucose transporter in *Komagataeibacter* strains may be the homolog of OprB porin from *Pseudomonas aeruginosa* (Wylie & Worobec, 1995). OprBs of *Komagataeibacter* are also likely beta‐barrel proteins (predictions made using Boctopus2). The number of *oprB* gene copies varies depending on genome, with the highest number of fifteen genes predicted for *K. sp*. SXCC1 strain (Supporting Information Figure S9). Due to the high number of *oprB* gene copies in each *Komagataeibacter* genome, it is possible that these proteins play an important role in carbohydrates transport (potentially, other than glucose as well; Wylie & Worobec, 1995). The conservation pattern of OprB orthologs highlights their diversity in the *Komagataeibacter* genus (Figure 4a).

Transport of glucose through the cytoplasmic membrane in bacteria most commonly include phosphoenolpyruvate‐dependent sugar phosphotransferase system (PTS), transporters dependent on proton or cation gradient, diffusion facilitators, or ATP‐driven ABC transporters (Jahreis, Pimentel‐Schmitt, Brückner, & Titgemeyer, 2008). Blastp search did not result in finding of homologs of *E. coli and V. furnissii* glucose‐PTS and mannose‐PTS systems’ components in any of the *Komagataeibacter* strains. Based on Prokka annotation and InterProScan search, we detected only the EIIA component of fructose‐specific PTS system. This gene is present in all the *K*. *xylinus* strains and is clustered together with several other components of PTS system (Supporting Information Figure S10). However, the genomes lacked other domains of the enzyme II complex (such as IIB, IIC, IID; Saier & Reizer, 1992), which may suggest that this system does not function in fructose transport. We continued blastp‐based search using sequences of known glucose transporters of *E. coli* (the methyl‐galactoside permease MglBAC and the galactose permease GalP of *E. coli*; the sodium‐coupled glucose permease GLT of *V. parahaemolyticus*; the glucose facilitator, Glf of *Z. mobilis*; glucose transport protein GlcP of *S. scabiei*) and found only homologs of the galactose permease GalP (Hernández‐Montalvo et al., 2003). Up to four copies of *galP* gene are present in the *Komagataeibacter* genomes and their products are well conserved (Figure 4b). Summarizing, our findings suggest, that glucose is not phosphorylated during transport through the inner membrane, but in the cytosol (by glucokinase).

The conservation pattern of both, OprB and GalP proteins, is almost exactly the same in *K. xylinus* E25, *K. xylinus* E26, *K. xylinus* BCRC 12334 strains. Furthermore, direct genome sequence comparison between these three strains did not identify a putative transporter present uniquely in *K. xylinus* E26 strain. Therefore, we cannot explain, at this stage, why *K. xylinus* E26 strain is more efficient in glucose‐based cellulose synthesis. It is therefore possible that the observed differences in cellulose synthesis yield between the three strains are not due to differences in glucose transport, but due to distinctive modes of glucose metabolism regulation.

#### 3.4.3 Predicted glycerol metabolic enzymes

It has been shown in *Escherichia coli* that the proteins encoded by the *glp* regulon mediate the utilization of glycerol and sn‐glycerol‐3‐phosphate (G3P) (Cozzarelli, Freedberg, & Lin, 1968). This regulon comprises of five operons which are located in three different regions of the genome (Zhao et al., 1994). We found homologs of *glp* proteins, which form one putative operon composed of *glpD*,* glpK*,* glpX*,* fba* genes in the tested *Komagataeibacter* strains (Figure 4c). The components of this operon encode: aerobic G3P dehydrogenase, a cytoplasmic glycerol kinase, fructose‐1,6‐bisphosphatase, fructose‐bisphosphate aldolase, respectively. The sequence upstream of the *glp* operon encodes the *glpF* gene, whose product is a cytoplasmic membrane protein facilitating diffusion of glycerol into the cell (Weissenborn, Wittekindtn, & Larsonsii, 1992). Adjacent to the *glp* operon is located the *glpR* gene, which encodes the repressor of the *glp* regulon of the *deoR* family of the transcriptional regulators (Zeng, Ye, & Larson, 1996). The repression of the *glp* regulon is relived in the presence of glycerol‐P (Zeng et al., 1996). The majority of *Komagataeibacter* genomes also contain a homolog of the *glpQ* gene (located outside of the *glp* operon), whose product is a periplasmic glycerophosphodiester phosphodiesterase. Moreover, several of the genomes carry additional copies of the predicted *glp* genes (Figure 4d). The structure of the *glp* operon is well conserved in the *Komagataeibacter* genomes, with the highest divergence displayed by the *K*. *hansenii* strains (Figure 4c). It can be noticed that, the *glpF* gene is much shorter in *K. xylinus* E25 strain than in other *K*. *xylinus* strains (Figure 4c). It is due to a single‐nucleotide insertion in its sequence, resulting in a frameshift and a premature termination (data not shown). Since GlpF is a putative glycerol diffusion facilitator, its disruption may negatively influence the glycerol uptake. This could partially explain, why *K. xylinus* E25 strain has the worst cellulose productivity out of the four in‐house strains, in the medium containing glycerol as carbon source (Figure 1b). On the other hand, a good performance of *K. hansenii* ATCC 53582 in this medium may be due to the presence of an additional copy of *glpF* gene in its genome (Figure 4d). A further inspection of the nucleotide sequence of the *glp* regulon allowed the detection of a polymorphism in the otherwise well‐conserved region of the *glpR* gene (Figure 4e). The *glpR* sequence of *K. xylinus E26* strain contained a six‐nucleotide deletion and several mutations, which translated to the deletion of two amino acids (Leu61 and Ala62 in the consensus sequence; Supporting Information Figure S11) and four amino acid substitutions (Ser63Gln, Ser64Gln, Ser71Pro, and Gly156Arg in the consensus sequence; Supporting Information Figure S11). The deletion and the adjacent mutations are located at the C‐terminus of the DeoR‐like helix‐turn‐helix DNA binding domain predicted in this protein (InterProScan analysis). It is possible that binding of the GlpR repressor to the operator of the *glp* operon is impaired in *K. xylinus* E26 strain. Therefore, the expression of the *glp* operon may be active even in the absence of glycerol. This may explain why *K. xylinus* E26 strain has the highest cellulose yield in the medium containing glycerol among the tested strains (Figure 1b). From a more general point of view, this result shows that such an important phenotypic difference may be due to discrete changes in a single gene sequence, which influences indirectly the metabolic pathways via regulatory mechanisms.

#### 3.4.4 Acetan biosynthesis gene cluster

**Figure 5:**
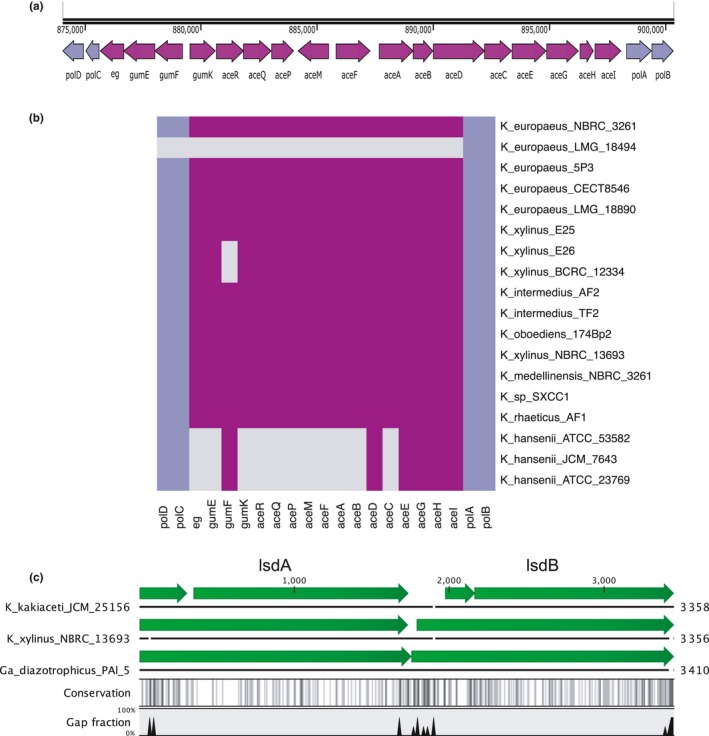
Conservation and characteristics of the soluble EPS gene clusters. (a) Organization of the acetan gene cluster in *K. xylinus* E25. Figure produced using SnapGene and modified. (b) Presence (magenta)/absence (gray) map of the acetan gene cluster and pol genes (navy‐blue). The gene symbols relate to putative functions: *polD (rmlD)*—dTDP‐4‐dehydrorhamnose reductase; *polC (rmlC)*—dTDP‐4‐dehydrorhamnose 3,5‐epimerase; e.g.,—endoglucanase; *gumE*—polymerization or export protein; *gumF*—acyltransferase; *gumK*—glucuronosyltransferase; *aceR*—rhamnosyl transferase; *aceQ*—glucosyl transferase; *aceP*—glucosyl transferase; *aceM* (*ugd*)—UDP‐glucose 6‐dehydrogenase; *aceF* (*mpg*)—mannose‐phosphate‐guanyl transferase; *aceA* (*gumD*)—UDP‐glucose:undecaprenyl‐phosphate glucose‐1‐phosphate transferase; *aceB* (*gumM*)—glycosyltransferase; *aceD* (*gumC*)—putative tyrosine‐protein kinase; *aceC* (*gumH*)—glycosyltransferase; *aceE*—polysaccharide transporter or flippase; *aceG*—putative beta‐barrel porin 2 family protein; *aceH* (*gumB*)—polymerization and export protein; *aceI*—acyltransferase; *polA* (*aceJ; rmlB*)—dTDP‐glucose 4,6‐dehydratase; *polB* (*rmlA*)—glucose‐1‐phosphate thymidylyltransferase. Annotation is based on homology with *gum*‐like gene cluster in *Kozakia baliensis* and acetan biosynthesis cluster from *K. sucrofermentans* DSM 15973 (Brandt et al., 2016; Ishida, Sugano, & Shoda, 2002). (c) Organization and conservation of the levan operon in *K. kakiaceti* JCM 25156, *K. xylinus* NBRC 13693, and *Ga. diazotrophicus* PAl 5. The gene symbols relate to putative functions: *lsdA*—levansucrase precursor; *lsdB*—levanase precursor. Annotation based on sequence homology with *Ga. diazotrophicus* SRT4's genes. Figure generated using CLC Sequence Viewer

*Komagataeibacter* species harbor the acetan biosynthesis cluster. These genes are homologous to *gum*‐like heteropolysaccharide genes of *Xanthomonas campestris* responsible for xanthan synthesis, which has been recently discovered in *Kozakia baliensis* (Brandt, Jakob, Behr, Geissler, & Vogel, 2016). In *K. xylinus* E25 strain, this cluster consists of seventeen genes (Figure 5a). It is completely absent in *K. europaeus* LMG 18494, whereas the majority of these genes is missing in the *K. hansenii* strains, which was additionally verified using tblastn (Figure 5b). The most important seems to be the absence of *aceA* (*gumD)*, which is thought to initiate acetan biosynthesis by transferring a glucosyl‐1‐phosphate residue from UDP‐glucose to an undecaprenyl‐phosphate lipid carrier anchored in the inner membrane (Brandt et al., 2016; Ishida, Sugano, & Shoda, 2002). This would implicate that the *K. hansenii* strains and *K. europaeus* LMG 18494 should not produce acetan. However, it has been shown that some strains of these species do secrete EPS of similar monosaccharides composition as in acetan (Fang & Catchmark, 2014, 2015; Valepyn, Berezina, & Paquot, 2012). It has been also observed that the presence of acetan in a culture medium influences its viscosity, thus enhancing cellulose dispersion (Ishida, Sugano, Nakai, et al., 2002). Moreover, it has been shown that EPS can modulate bundling and width of cellulose ribbons, and thus influencing cellulose porosity (Fang & Catchmark, 2014, 2015). Therefore, the differences in cellulose membrane structure observed for *K. hansenii* and the other three in‐house strains may be due to the divergence in soluble EPS synthesizing enzymes.

The polymerization and export of acetan in the *K. xylinus* species seems to follow the Wzx/Wzy pathway due to the presence of *aceD*,* aceE*,* aceG*, and *aceH* genes, which putatively code for polysaccharide copolymerase (PCP), flippase, beta‐barrel porin, and outer membrane transport protein (OPX), respectively (Schmid, Sieber, & Rehm, 2015). Interestingly, these genes are among the few conserved in the *K. hansenii* strains. Genomic analysis of the neighborhood of these genes, in the *K. hansenii* strains, revealed presence of several putative and unknown glycosyltransferases (data not shown). It is possible that the *K. hansenii* strains synthesize other than acetan, yet an uncharacterized EPS.

The acetan gene cluster is flanked by *pol* genes (colored in navy blue in the Figure 5a,b). These genes are organized in a cluster in *Acetobacter tropicalis* and *Kozakia baliensis* (*polABCDE*; Deeraksa et al., 2005; Brandt et al., 2016). This cluster is responsible for the biosynthesis of capsular polysaccharide (CPS), which consists of galactose, glucose, and rhamnose. It has been shown that *polE* plays an important role in this process, since it is likely responsible for anchoring of the polysaccharide to the cell surface (Deeraksa et al., 2005). CPS is thought to serve as a protective barrier from acetic acid in AAB. However, it has been observed that *Komagataeibacter* species do not produce this membrane polysaccharide (Andrés‐Barrao et al., 2013; Barja et al., 2016). Our genomic analysis supports this finding as we observed that in all analyzed strains, *polABCD* operon is disrupted by the acetan cluster and, moreover, lacks the crucial *polE* gene (Figure 5a,b). *Komagataeibacter* species must clearly have a distinctive, from other AAB, mechanism for acetic acid resistance.

#### 3.4.5 Levan biosynthesis

Apart from acetan, *K. xylinus* was also reported to synthesize levan, an EPS produced from extracellular sucrose (Kornmann et al., 2003; Limoli, Jones, & Wozniak, 2015). Activity of levansucrase, which is the main enzyme involved in the levan biosynthesis process, has been reported in *K. xylinus* I‐2281 strain (Kornmann et al., 2003). By screening *Komagataeibacter* genomes (using tblastn with the levansucrase (LsdA) protein sequence of *Ga. diazotrophicus* SRT4; Arrieta et al., 2004), we were unable to find levansucrase gene in all but *K. xylinus NBRC* 13693 and *K. kakiaceti* JCM 25156 strains. In *K. xylinus NBRC 13693* strain, levansucrase gene is arranged in a cluster with levanase (*lsdB*) gene (Figure 5c) and the components of the type II secretion system (data not shown), similarly as it has been reported in *Ga. diazotrophicus* (Arrieta et al., 2004). The levanase is responsible for hydrolysis of levan to free fructose. Therefore, these genes are necessary for the bacterium to feed on sucrose. It has been reported that in *Gluconacetobacter* representatives, sucrose cannot be transported through cell membrane and has to be hydrolyzed into glucose and fructose in the periplasm (Velasco‐Bedrán & López‐Isunza, 2007). However, the obtained results suggest that the majority of *Komagataeibacter* strains should not synthesize levan. Other systems of sucrose transport and hydrolysis were not reported for this genus, and we were unable to discover them in the sequenced genomes. We have shown that culturing of the in‐house strains in the medium with sucrose results in a low cellulose yield, except for *K. xylinus* BCRC 12334 strain (Figure 1b). Since the genome of *K. xylinus* BCRC 12334 strain does not encode the *lsdA‐lsdB* operon, this would suggest that there should exist another, yet undefined, system for sucrose metabolism in *Komagataeibacter* strains.

#### 3.4.6 Conservation of cellulose synthase operons’ structure

**Figure 6:**
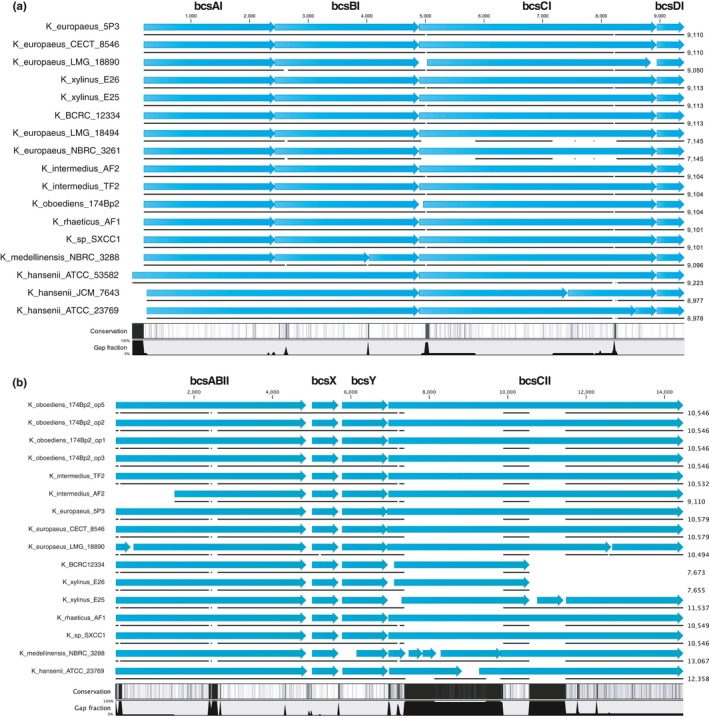
Comparison of cellulose synthase operons’ structure and nucleotide sequence among the *Komagataeibacter* strains. Operons contained within a single contig or a chromosome are only shown. (a) Operon type I. (b) Operon type II. Figures generated using CLC Sequence Viewer and modified

In *Komagataeibacter*, cellulose synthase enzyme is encoded by two types of operons (Matsutani et al., 2015). The type I *bcs* operon consists of four genes: *bcsAI, bcsBI*,* bcsCI*, and *bcsDI* (Matsutani et al., 2015). BcsA is a β‐glycosyltransferase, an inner membrane protein, which catalyzes the synthesis of β‐1,4‐glucan from UDP‐glucose (Jedrzejczak‐Krzepkowska et al., 2016; Morgan et al., 2016). Furthermore, PilZ domain of this subunit has been shown as c‐di‐GMP binding site (Fujiwara et al., 2013; Morgan, Strumillo, & Zimmer, 2012). BcsB is a periplasmic protein, which participates in β‐glucan chain synthesis and translocation (Jedrzejczak‐Krzepkowska et al., 2016). Role of BcsC and BcsD is unclear, and however, they are required for cellulose biosynthesis *in vivo* (Jedrzejczak‐Krzepkowska et al., 2016; Römling, 2002). The type I cellulose synthase operon has on average size of 9 kbp (Figure 6a). When the structure of this operon is compared inside of the *Komagataeibacter* genus, several differences can be observed. First, in the *K. hansenii* strains, genes coding for subunits *bcsAI* and *bcsBI* are fused, unlike in the *K. xylinus* strains, as it was reported before (Saxena et al., 1994). What is more, some diversity in the structure of *bcs* genes can be observed. The subunit A of *K. hansenii* ATCC 53582 strain is much longer than in any of the *Komagataeibacter* strains. For *K. hansenii* ATCC 23769 and *K. hansenii* JCM 7643 strains, *bcsCI* is split into two, not similar parts. In case of other strains, for *K. medellinensis*,* bcsBI* is disrupted due to a frameshift mutation, as previously reported for this cellulose‐negative strain (Matsutani et al., 2015). The alignment shown in the Figure 6a highlights a large deletion in the sequence of *bcsCI* in two *K*. *europaeus* strains, LMG 18494 and NBRC 3261. Overall, the *bcs* operons of the *K. *xylinus E25, E26, and BCRC 12334 strains are very similar to those of the *K*. *europaeus* strains. Interestingly, one subunit that is very well conserved among *Komagataeibacter* strains is *bcsDI,* which function is up to date the most speculative. It was suggested that *bcsDI* has originally evolved in AAB since it lacks homology with any other bacteria (Matsutani et al., 2015).

The type II *bcs* operon consists of four genes, *bcsABII*,* bcX*,* bcsY*, and *bcsCII*. It is believed that *bcsII* synthesizes the acylated, amorphous cellulose due to homology of *bcsY* to transacylase (Chawla et al., 2009; Umeda et al., 1999). This operon is absent in *K. europaeus* LMG 18494 and *K. europaeus* NBRC 3261 strains, as well as in *Ga. diazotrophicus* PAl 5 strain (Figure 6b), which was additionally checked using tblastn. *K. oboediens* 174Bp2 is unusual, as its genome contains four copies of *bcsII* operon (Figure 6b). In other *Komagataeibacter* strains, *bcsCII* is often disrupted, as in the case of *K. europaeus* LMG 18890, *K. xylinus* E25, *K. medellinensis* NBRC 3288, and *K. hansenii* ATCC 23769 strains. In *K. xylinus* E25 and *K. medellinensis* NBRC 3288 strains, this disruption is caused by an insertion sequence, as it was previously reported (Matsutani et al., 2015). Additionally, there are many insertions or deletions (InDels) present at the start of the *bcsCII* across the compared strains, likely causing frameshift mutations in some of them.

Sequenced‐based predictions suggest that *bcsCII*, like *bcsCI*, is a beta‐barrel protein, likely forming a channel in the outer membrane (predictions made using Boctopus2; Hayat, Peters, Shu, Tsirigos, & Elofsson, 2016). Since the putative role of *bcsC* is export of cellulose, disturbance of *bcsCII* may influence secretion of the acylated polymer. Furthermore, sequence variations, in the both subunits, across *Komagataeibacter* strains may be responsible for differences in cellulose structure. Generally, the often‐seen disruption of mainly the *bcsC* subunits suggests that cellulose export may be the first target of evolutionary forces.

#### 3.4.7 Diversity in the c‐di‐GMP‐based regulatory network

Cyclic di‐GMP ubiquitous second messenger, until recently regarded as bacteria‐specific (Hengge, Gründling, Jenal, Ryan, & Yildiz, 2016), crucial for life style and cell cycle regulation was discovered in *K. xylinus* in late 1990s, but elucidation of complexity of its signaling network was done in other species (Tal et al., 1998). Most frequently c‐di‐GMP signaling is being proved to be involved in the regulation of soluble and insoluble EPS components production during biofilm formation and dispersion (Römling & Galperin, 2015 and references therein). Typically, c‐di‐GMP regulatory networks are composed of numerous enzymes catalyzing its synthesis (diguanylate synthases with DGGEF domains, DGCs) and hydrolyzing it into linear pGpG (phosphodiesterases with EAL domains, PDEs) or directly into two molecules of GTP (phosphodiesterases with HD‐GYP domains). Complexity of this network may be illustrated by the number of genes involved: from around 20 in, for example, *Escherichia coli* K‐12 (29 genes; Povolotsky & Hengge, 2016) or alpha‐proteobacterium *Sinorhizobium meliloti* (22 genes; Schäper et al., 2015), up to dozens, for example, in *Pseudomonas aeruginosa* (42 genes; Valentini & Filloux, 2016) or in alpha‐proteobacteria *Bradyrhizobium japonicum* (55 genes; Schäper et al., 2015). Besides enzymatic activity, GGDEF/EAL proteins frequently serve as signal receiving proteins via their sensory domains (e.g., PAS, GAF, CHASE or BLUF; Römling & Galperin, 2015; Hengge et al., 2016). Complete understanding of biological effect regulated by c‐di‐GMP includes identification of effector molecules containing riboswitches and diverse proteins capable of c‐di‐GMP molecule binding (most frequently but not exclusively via PilZ domains; Hengge et al., 2016). Since pioneering work of Prof. Benziman's group resulting in discovery of c‐di‐GMP and three operons composed of pairs of DGC and PDEs (*cdg*1‐3; Tal et al., 1998), no further elucidation of this signaling network in any *Komagataeibacter* strain has been published. Therefore, we decided to take advantage of the available genomic data to shed some light into level of complexity of this important regulatory network in the tested genus. Furthermore, analysis presented here suggests that source of phenotypic diversity observed among in‐house strains does not stem from any large changes in the genome content, but rather from discrete changes, outside of the main metabolic pathways (e.g., the glycerol repressor in *K. xylinus* E26 strain).

**Figure 7:**
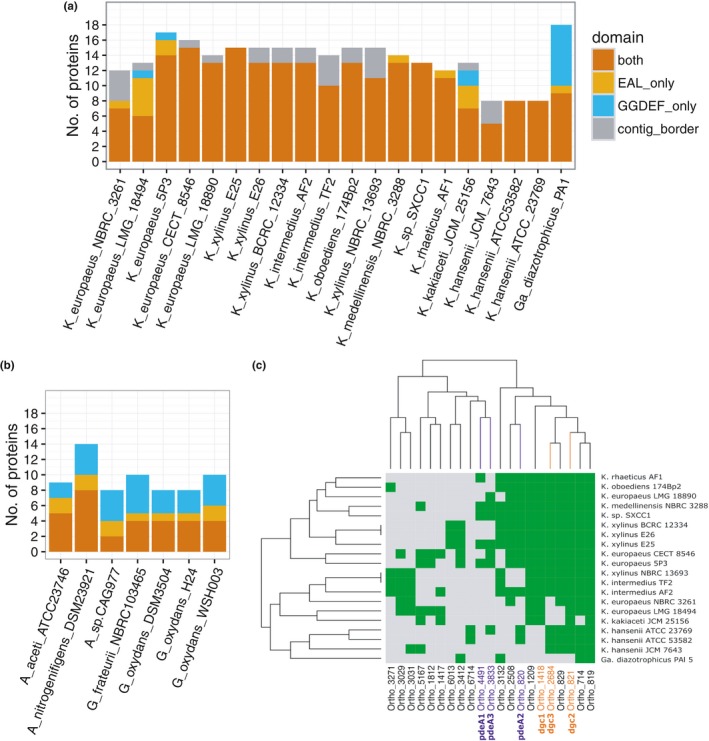
Characteristics and conservation of c‐di‐GMP‐metabolizing proteins. (a) Number of GGDEF/EAL domain‐containing proteins in genomes of *Komagataeibacter* spp. and *Gluconacetobacter diazotrophicus* PAl 5. Distinguished are the proteins containing both domains (in red) or only one (EAL in yellow; GGDEF—in blue). In gray are given single‐domain proteins encoded by genes that are located at contigs borders and therefore are of lower reliability (see Methods). (b). Genomes of the *Acetobacter* (A) and the *Gluconobacter* (G) genus. The strains were selected based on the completeness of their genome sequence (full genome, or less than 50 contigs). (c) Presence (green)/absence (gray) map of GGDEF/EAL domain‐containing proteins in the analyzed genomes. Orthologs of diguanylate cyclases (*dgc*) and phosphodiesterases (*pde*) identified earlier (Tal et al., 1998) are colored in orange and dark blue respectively. Dendrogram was generated based on hierarchical clustering analysis. *Y*‐axis: strains clustering; *x*‐axis: protein clustering

By running InterProScan on the entire predicted proteomes of *Komagataeibacter* strains, we selected proteins, which matched EAL domain or/and GGDEF domain pattern. In the majority of proteins, both domains were present in one protein. Number of EAL/GGDEF proteins varied, with the *K. xylinus* clade genomes having up to seventeen and the *K. hansenii* clade harboring consistently eight proteins (Figure 7a). Already at this point, it appears a unique feature of the *Komagataeibacter* genus to have both domains present in tandem in the majority of c‐di‐GMP‐metabolizing proteins (Figure 7a). In *Gluconacetobacter diazotrophicus* PAl 5 and in strains of genera *Acetobacter* and *Gluconobacter,* it appears that this distribution is less skewed and there are many proteins harboring only a single, mostly a GGDEF, domain (Figure 7a,b). These numbers are nevertheless much higher than previously proven by Prof. Moshe Benziman (three *cdg* operons mentioned above; Tal et al., 1998).

After observing diversity in the number of c‐di‐GMP turnover proteins across the *Komagataeibacter* genomes, we next investigated how well these proteins are conserved. We observed a very complicated presence/absence pattern of these important proteins in the twenty analyzed genomes (Figure 7c). Furthermore, even the three canonical operons identified by Tal et al. (1998) turned out not to be well conserved (highlighted in blue [PDEs] and in orange [DGCs]). Surprisingly, PdeA1 and Dgc1, the most highly expressed proteins in *K. xylinus* strains tested in pioneering studies (in original paper: *A. xylinum* strains: 1306‐3, an isolate from strain B42; North Regional Research Laboratories, Peoria, Ill; Tal et al., 1998), are both absent in the genomes of the *K. hansenii* strains. Moreover, diguanylate cyclases (*dgc1‐3*) seem to be better conserved than phosphodiesterases (*pdeA1*,* pdeA3*). These observations are drawn from the analysis based on mostly draft genomes and therefore have to be treated cautiously. For example, for both, the *K. xylinus* BCRC 12334 and *K. xylinus* E26 genome, we checked, using tblastn, that these genomes may indeed contain the *pdeA1* and *pdeA3* genes, but at contig borders. This may likely be the explanation of this surprising incompleteness of these important operons, since the two complete genomes of *K. xylinus* E25 and *K. medellinensis* NBRC 3288 harbor them. On the other hand, the only closed *K. hansenii* genome sequence, ATCC 23769, is missing the *cdg1* operon, which would support the hypothesis that the c‐di‐GMP regulatory network have diverged in the *K. hansenii* strains, with respect to the *K. xylinus* strains. Furthermore, none of the *cdg* operons is conserved in *Ga. diazotrophicus* PAl 5 strain. Additionally, searching of orthologs of these genes in other AAB (as those shown in the Figure 7b) was similarly unsuccessful.

Fast evolution of c‐di‐GMP signaling network was previously observed in genomic comparison study of 61 pathogenic and commensal *Escherichia coli* strains (Povolotsky & Hengge, 2016). The authors identified numerous losses of DGCs besides many mutational changes in pathogenic strains when compared to nonviral strains. Therefore, we expect that the observed by us diversity in the atypical (as composed of only several and mainly two‐domain GGDEF/EAL proteins) c‐di‐GMP signaling network in *Komagataeibacter* species is of great importance for phenotypes of cellulose producers. Elucidation of biochemical role of the identified genes in *Komagataeibacter* strains and precise analysis of their possible interactions with EPS synthesis and secretion machineries as well as with other cellular processes should bring a valuable impact on productive strain engineering.

## 4 CONCLUSIONS

This first comparative genomic approach conducted by us in the *Komagataeibacter* genus brought several genomic evidences for a clear separation of two main clades, namely: of the *K. hansenii* strains and larger, of the *K. xylinus*‐related strains. This conclusion is based on whole genome sequence similarity comparisons measured using MUM index as well as through phylogenetic analysis employing 868 ortholog gene clusters. The distinctiveness of the *K. hansenii* strains was further supported by the pattern of intragenus distribution of the predicted mobile elements and the presence of different genome defense systems, that is, variable toxin‐antitoxin systems in the *K. xylinus* group and CRISPR‐Cas loci in the *K. hansenii* strains. Moreover, functional diversity analysis showed that genetic diversity between the two main *Komagataeibacter* clades is beyond the well‐known differences in cellulose operon organization and is manifested by, for example, lack of cluster of genes responsible for acetan synthesis and changes in the structure of the putative glycerol diffusion facilitator gene, as well as differences in size and composition of c‐di‐GMP signaling network (including absence of PdeA1 and Dgc1) in the *K. hansenii* strains. All these results taken together suggest that *K. hansenii* strains seem to form a subgenus, separated from all other strains tested here (named the *K. xylinus* clade).

Genomic diversity among *Komagataeibacter* strains, shown here on numerous examples, is particularly interesting from the practical point of view for two functional categories: EPS synthesis and c‐di‐GMP signaling network. Better characterization of soluble EPS synthesized, in parallel or alternatively to cellulose, in different *Komagataeibacter* strains may aid optimization of BNC productivity. Moreover, cellulose pellicle formation is a very precisely controlled process and involves molecular regulatory mechanisms such as c‐di‐GMP signaling. We predicted presence of several proteins with GGDEF and EAL domains in every *Komagataeibacter* strain. Such predominant presence of dual‐domain proteins is not usual in c‐di‐GMP signaling networks in AAB and may suggest primitive stage of these pathways organization in the *Komagataeibacter* genus. There is not enough data to speculate about specialization of the discovered genes in c‐di‐GMP metabolism or signaling by allosteric interactions. Nevertheless, cellulose producers seem to be a very poorly studied group of bacterial strains, which, when explored further, can bring interesting molecular discoveries in nucleotide‐regulated signaling field.

Importantly, this work contributed the first description of gene clusters important for carbohydrate metabolism (glucose and glycerol transport, EPS synthesis and secretion). Furthermore, comparative analysis enabled finding of *Komagataeibacter* features, believed to be genus‐typical, which appeared to be strain‐specific, for example, acetan and levan synthesis, or presence of three *cdg* operons. Therefore, many metabolic features, revealed by the previously reported production process optimizations, may appear not common for the whole genus. Moreover, great phenotypic differences may not be clearly distinguishable on genome level as it was exemplified here by our *K. xylinus* in‐house strains, which were overall similar in DNA sequence. Most probably phenotypic diversity among the compared strains was due to discrete sequence changes (*glpR* polymorphism in *K. xylinus* E26 strain) or fine‐tuning of gene expression and protein activities via yet to‐be‐discovered signaling pathways (the poorly understood c‐di‐GMP signaling network, hypothetical proteins identified as unique on genomic islands and in ortholog groups). Taken together, our findings showed that the genomic approach is sensible for elucidation of the numerous misleading results found in the literature. Precise description of any new strain, preferably together with genomic sequence, is of great importance for utility of published results concerning *Komagataeibacter* representatives. Phylogenetic studies, performed in this work, brought evidence for usefulness of Cleenwerck et al. method for intragenus classification of the *Komagataeibacter* strains and should be chosen for taxonomy verification of any new strain.

In the future, together with the growing number of genomic sequences and biochemical studies, new genome engineering strategies should become available for cellulose production optimization.

## CONFLICT OF INTEREST

The authors declare that they have no competing interests.

## AUTHORS CONTRIBUTION

MR, KK, and SB conceived and designed the study. KK, MJ‐K, and PJ cultured bacteria and isolated DNA. MR assembled and annotated the genomes. KK performed the bioinformatics analyses regarding IS, prophages, and GIs. MR performed the remaining bioinformatics analyses. MR and KK drafted the manuscript. All authors read and approved the final manuscript.

## ETHICAL STATEMENT

The presented research did not involve studies with human or animal subjects or recombinant DNA.

## Data Availability

The DNA sequences of the assembled genomes are available at NCBI under the BioProject accession: PRJNA339514, PRJNA339679, PRJNA339678. Additional data sets generated in this work are provided in the Supporting Information Data S1 of this article.
