# Supplementary material for: Comparative genomics of the Komagataeibacter strains—Efficient bionanocellulose producers
Source: Microbiologyopen. 2018 Oct 26;8(5):e00731. doi: 10.1002/mbo3.731 (PMC6528568; doi:10.1002/mbo3.731)
Supplement: Supplementary file 1 [file MBO3-8-e00731-s001.docx]

Supplementary Material

**Comparative genomics of the *Komagataeibacter* strains - efficient bionanocellulose producers**

**Małgorzata Ryngajłło^1*^, Katarzyna Kubiak^1^, Marzena Jędrzejczak-Krzepkowska^1^, Paulina Jacek^1^, Stanisław Bielecki^1^**

1 Institute of Technical Biochemistry, Lodz University of Technology, B. Stefanowskiego 4/10, 90-924 Lodz, Poland

* **Correspondence:**

Małgorzata Ryngajłło

malgorzata.ryngajllo@p.lodz.pl

**Supplementary Figures**


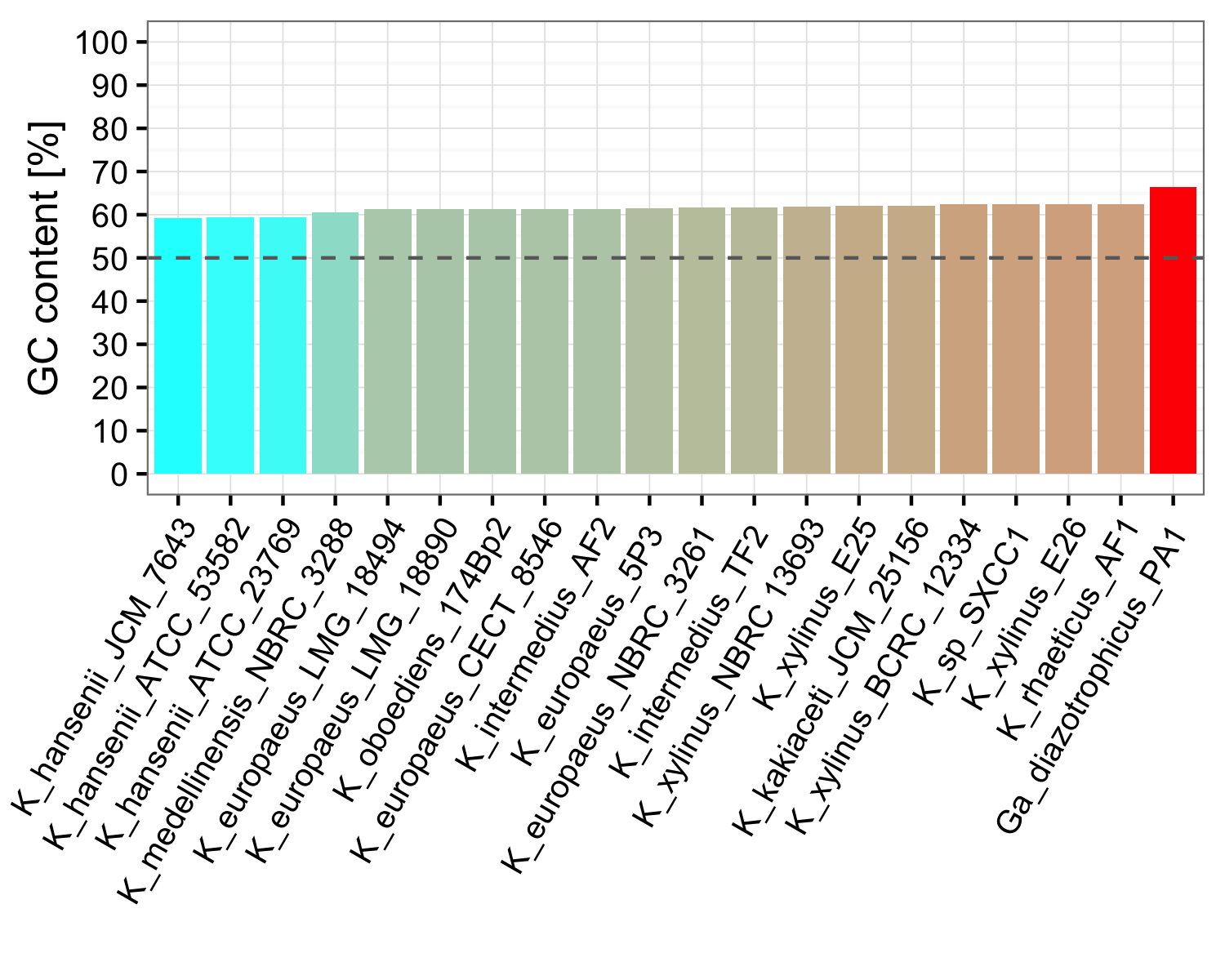


**Supplementary Figure 1**. Genome DNA G+C content of the nineteen *Komagataeibacter* strains and a *Ga. diazotrophicus* PAI 5. Horizontal dashed line marks the G+C content of 50%.


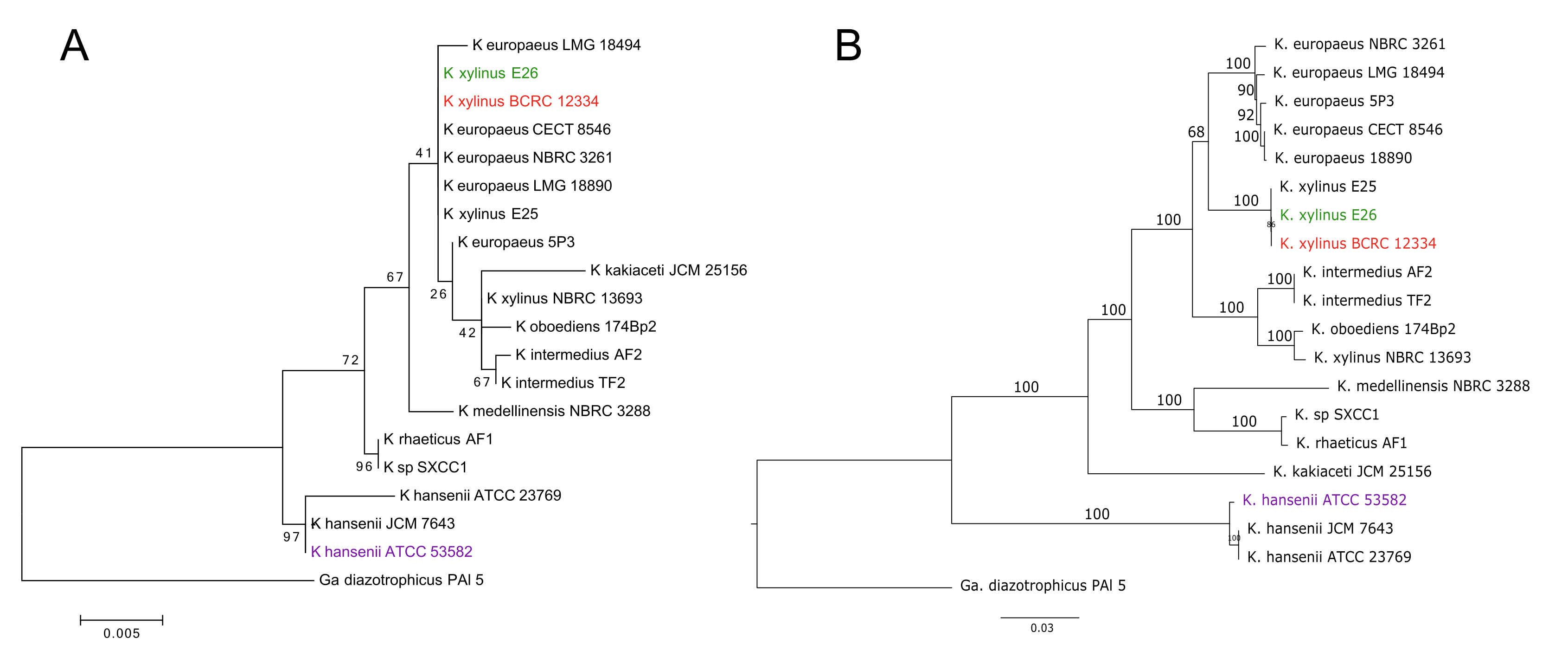


**Supplementary Figure 2**. Maximum Likelihood phylogenetic trees generated based on sequences of **A).** predicted 16S rRNA gene sequences, **B).** 3 housekeeping genes (dnaK, groEL, and rpoB). The numbers above branches represent percentage bootstrap values from 500 replications. Phylogenetic tree was constructed using MEGA (A) or RAxML (B). The bar represents 0.5% or 3% sequence divergence on A) and B), respectively.


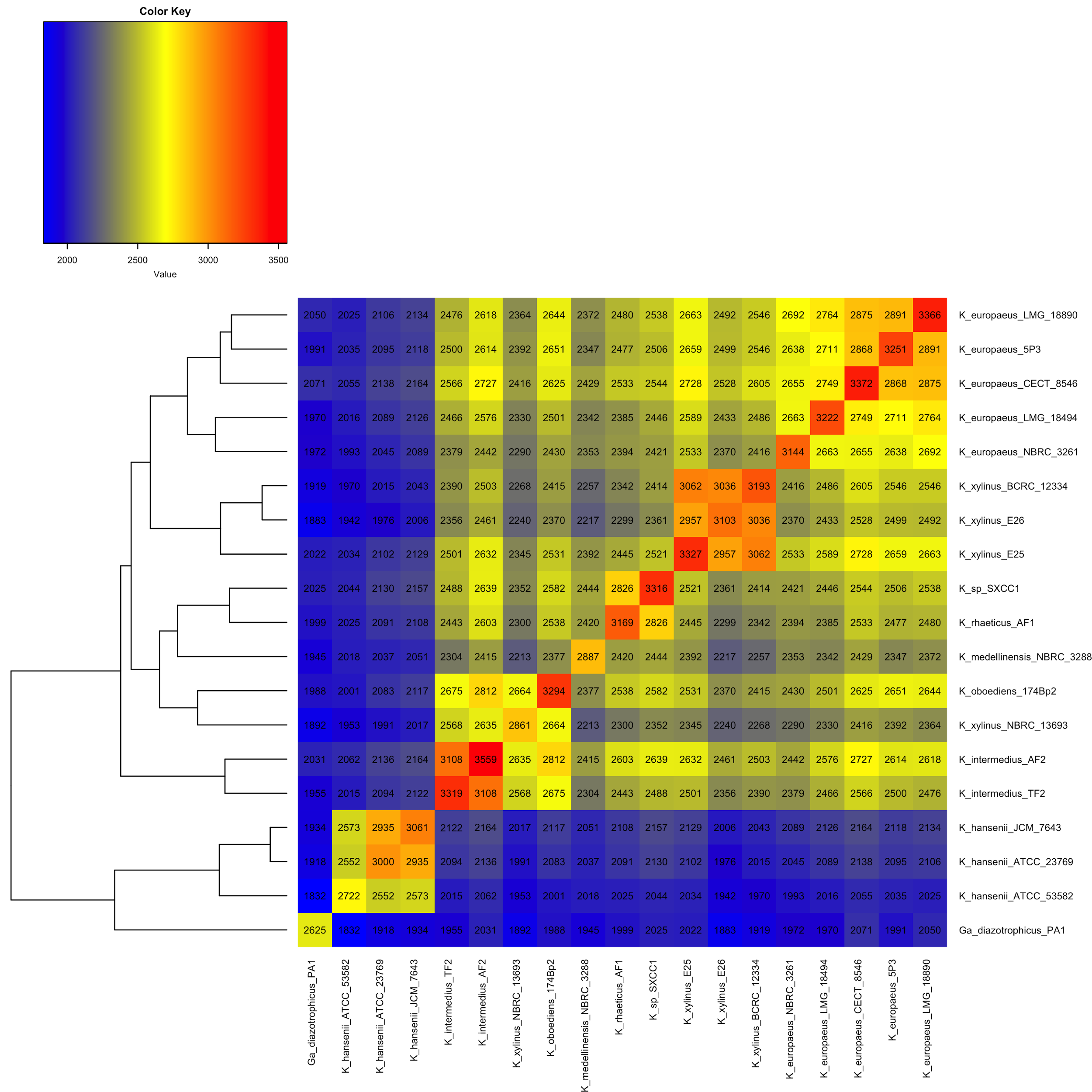


**Supplementary Figure 3.** Number of orthologs shared between twenty *Komagataeibacter* strains and *Ga. diazotrophicus* PAI 5.


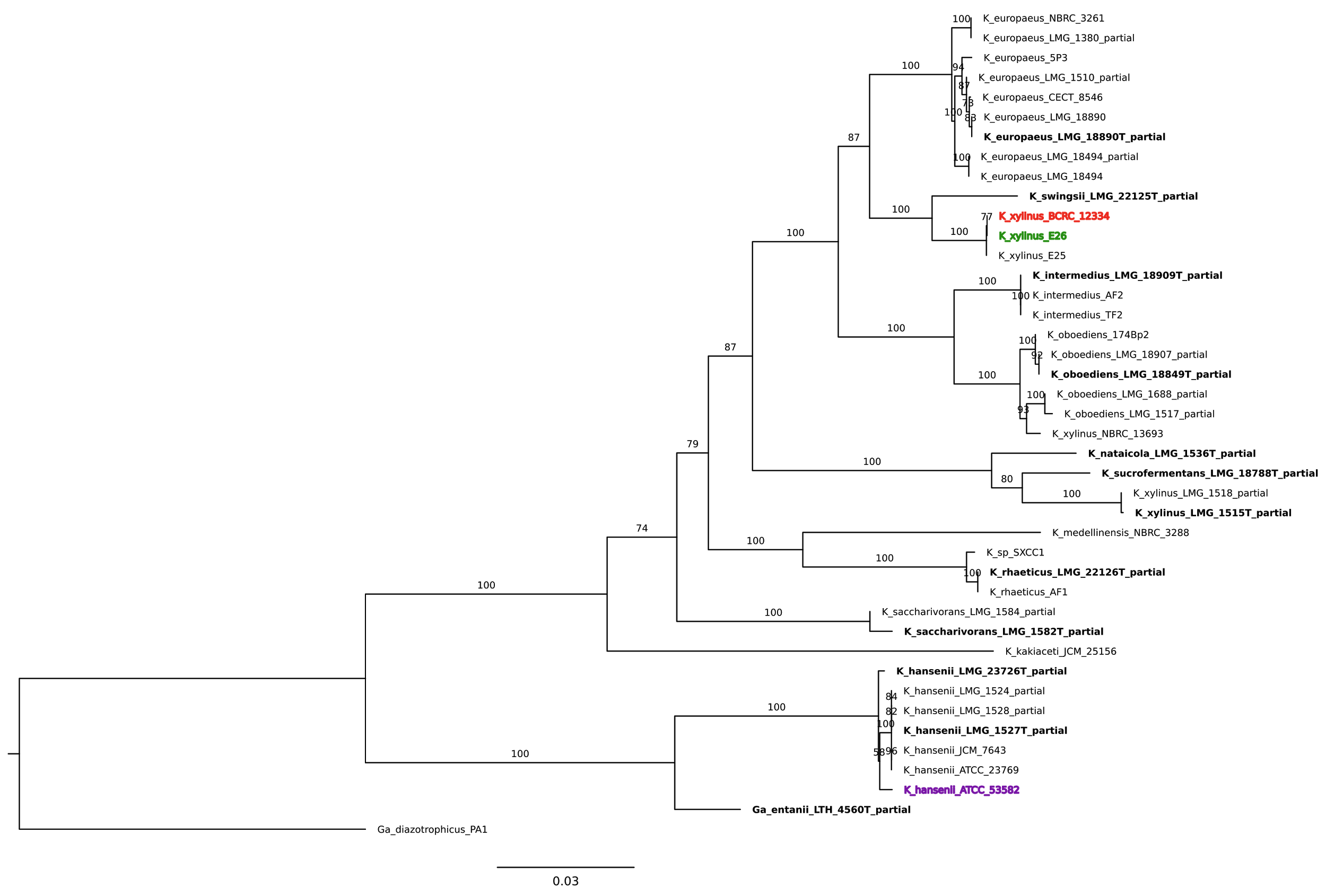


**Supplementary Figure 4**. Maximum Likelihood phylogenetic trees generated based on sequences of 3 housekeeping genes (*dnaK*, *groEL*, and *rpoB*). To construct the tree, complete sequences of the genes from the 20 analyzed in this work genomes were used jointly with partial sequences from the work of Cleenwerck et al., 2010 (denoted here with “partial” suffix). The type strains are given in bold. The numbers above branches represent percentage bootstrap values from 500 replications. Phylogenetic tree was constructed using RAxML. The bar represents 3% sequence divergence.


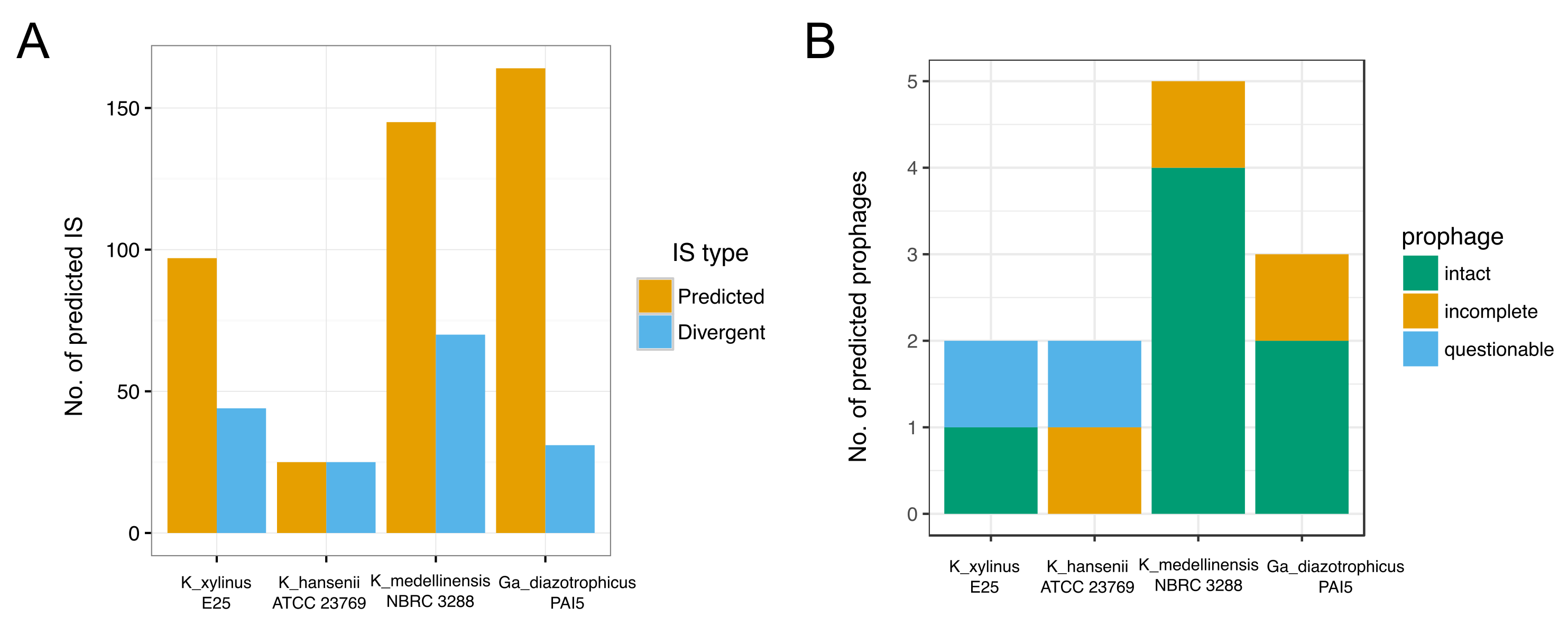


**Supplementary Figure 5.** Predicted mobile elements in chromosomes from two complete *Komagateibacter* genomes (*K. xylinus* E25 and *K. medellinensis* 3288 strains), chromosome sequence of *K. hansenii* ATCC 23769 strain and reference, free-living *Gluconacetobacter diazotrophicus* PAI 5 strain. **A).** Number of predicted (orange) and divergent (blue) insertional sequences annotated by ISsaga online tool. **B).** Number of prophage sequences identified as intact (green), questionable (blue) or incomplete (orange) by PHASTER online tool.


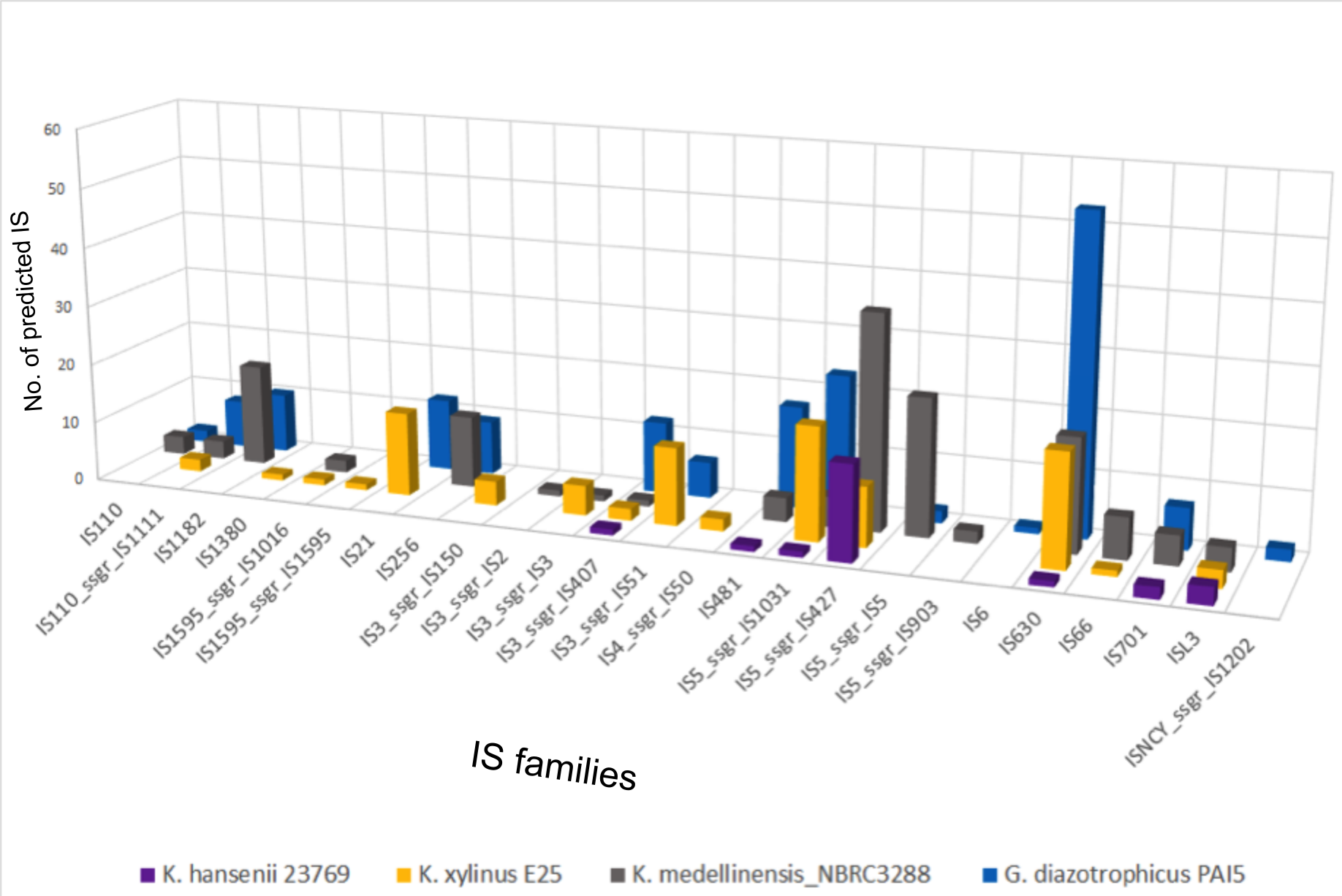


**Supplementary Figure 6.** Distribution and number of predicted IS from different families in the *K. xylinus* E25, *K. medellinensis* 2388, *K. hansenii* 23769 and *Ga. diazotrophicus* PAI 5 identified by ISSaga online tool.

**
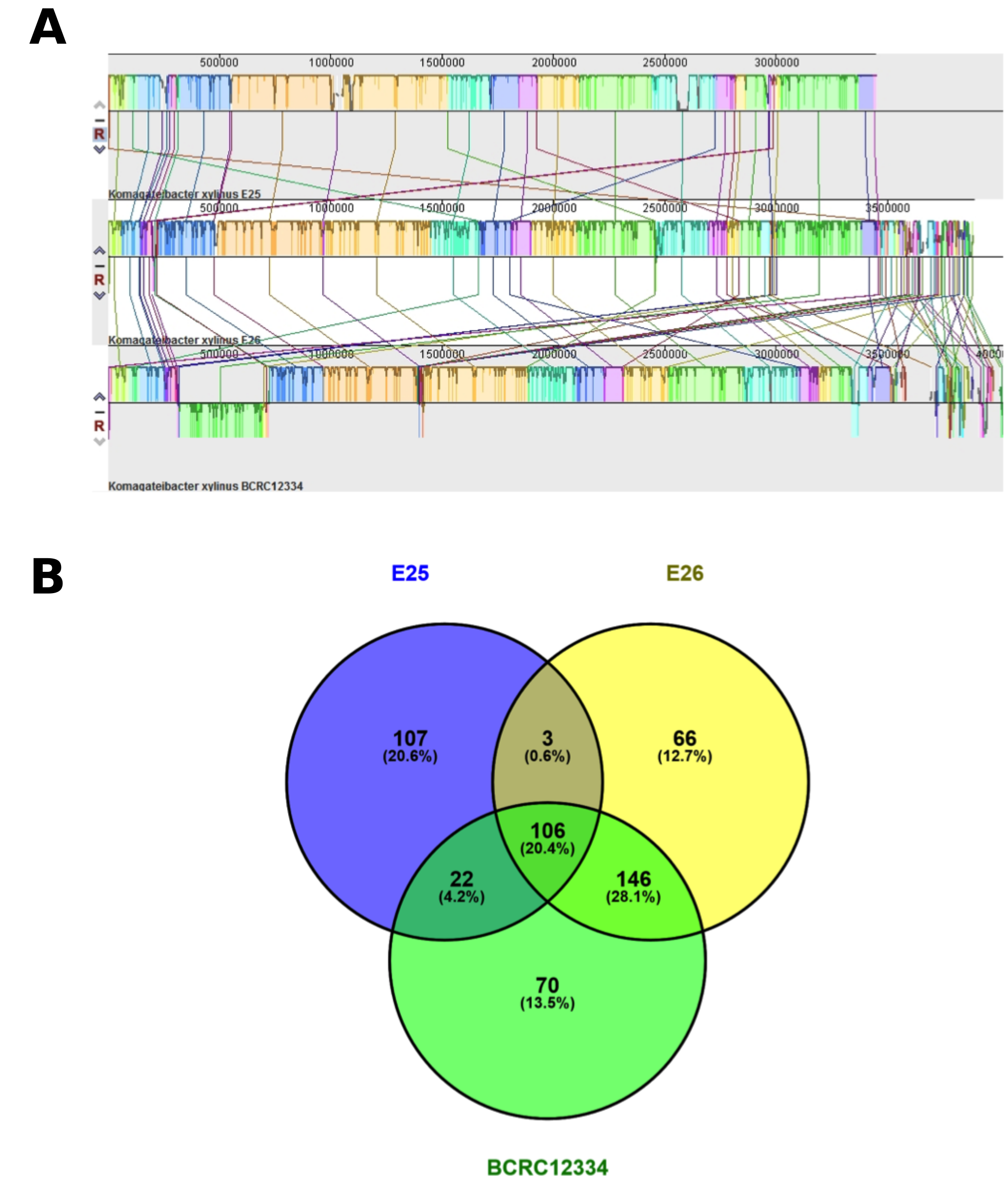
**

**Supplementary Figure 7.** Comparisons of genomic islands identified in the three sequentially most similar genomes: *K xylinus* E25, E26 and BCRC 12334 strains **A.** Sequence alignment generated in Mauve. **B.** Number of common and unique orthologs; Venn’s diagram prepared with Venny 2.1.


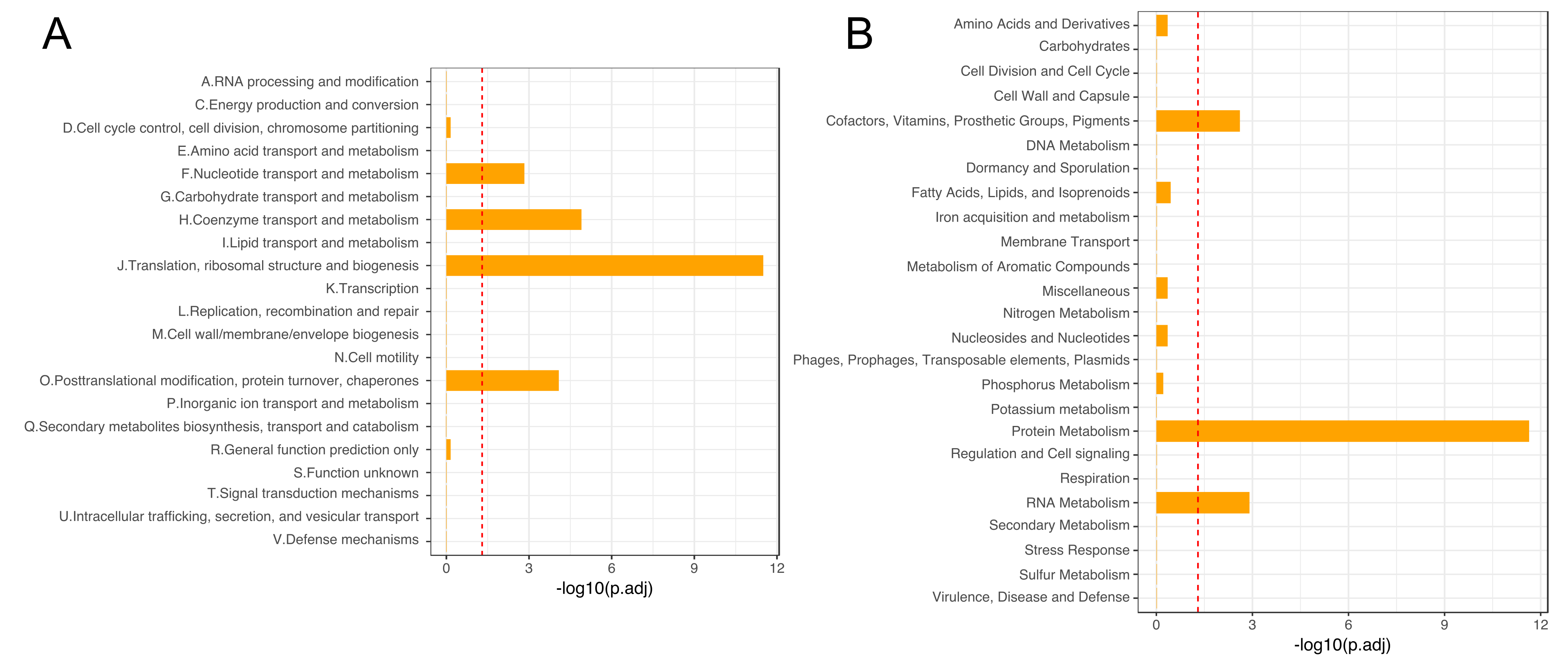


**Supplementary Figure 8.** Functional enrichment of the core genome. COG (A) and RAST (B) functional categories are displayed. Shown are results of one-sided Fisher’s Exact test (one-tailed). The red, dashed vertical line indicates adjusted p-value threshold of 0.01.


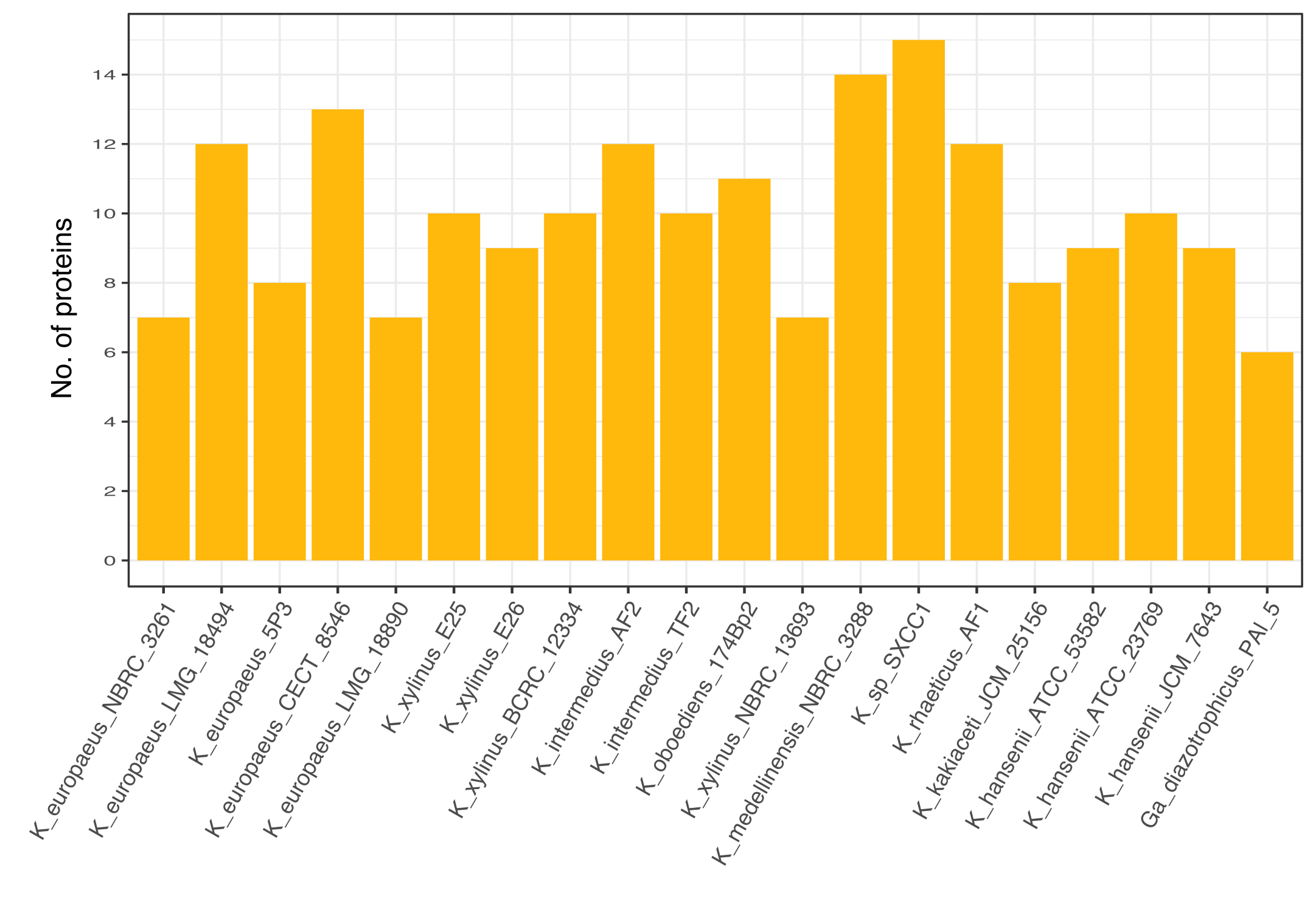


**Supplementary Figure 9**. Number of OprB homologs in the *Komagataeibacter* genomes and in the genome of *Ga. diazotrophicus* PAI 5.


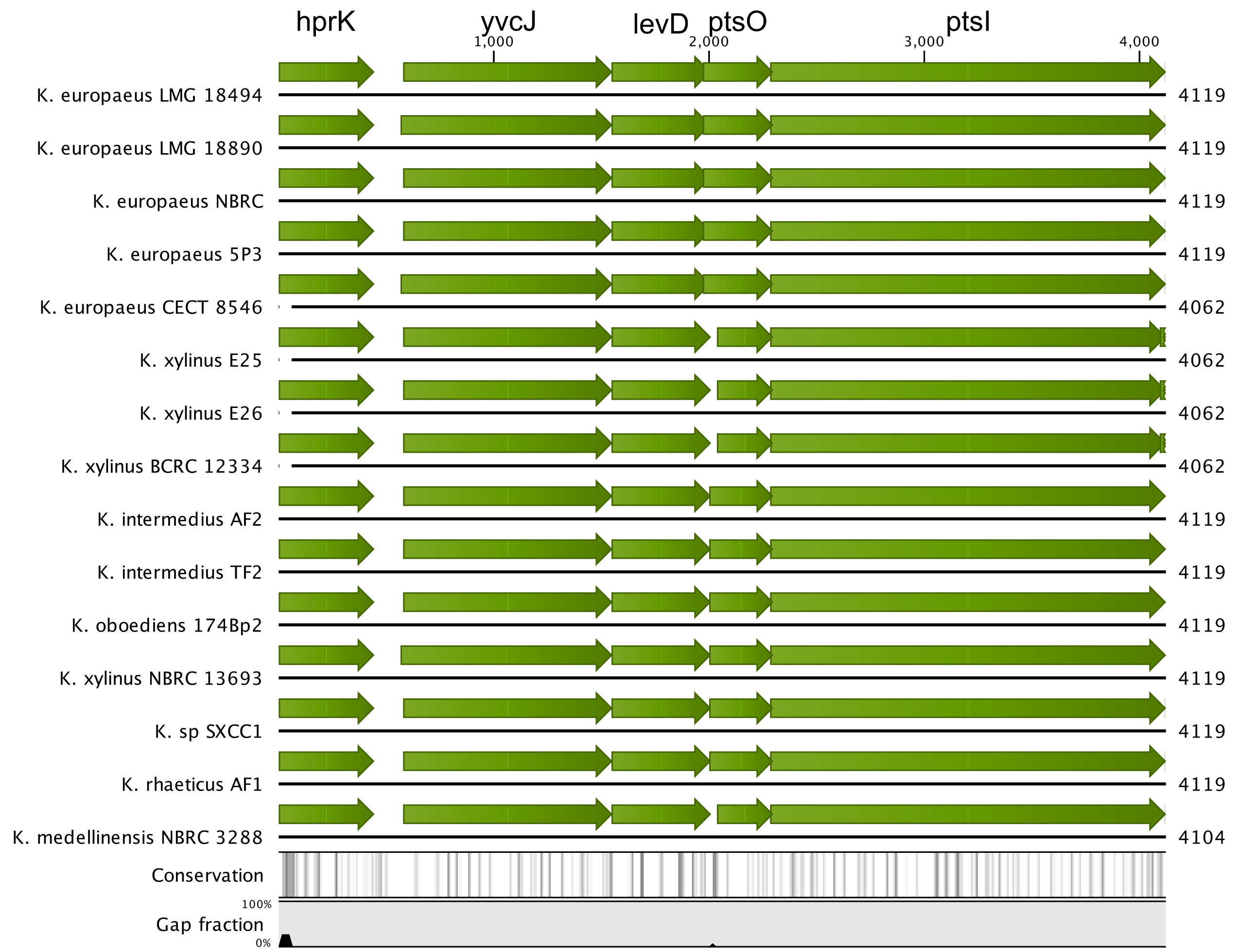


**Supplementary Figure 10**. Conservation of PTS system components in the *Komagataeibacter* genomes. The gene symbols relate to putative functions: *hprK* – Hpr kinase/phosphorylase; *yvcJ* – nucleotide-binding protein YvcJ; *levD* - PTS system fructose-specific EIIA component; *ptsO* – phosphocarrier protein NPr; *ptsI* - phosphoenolpyruvate-protein phosphotransferase. Annotation assigned by Prokka based on similarity to proteins from UniProtKB database.


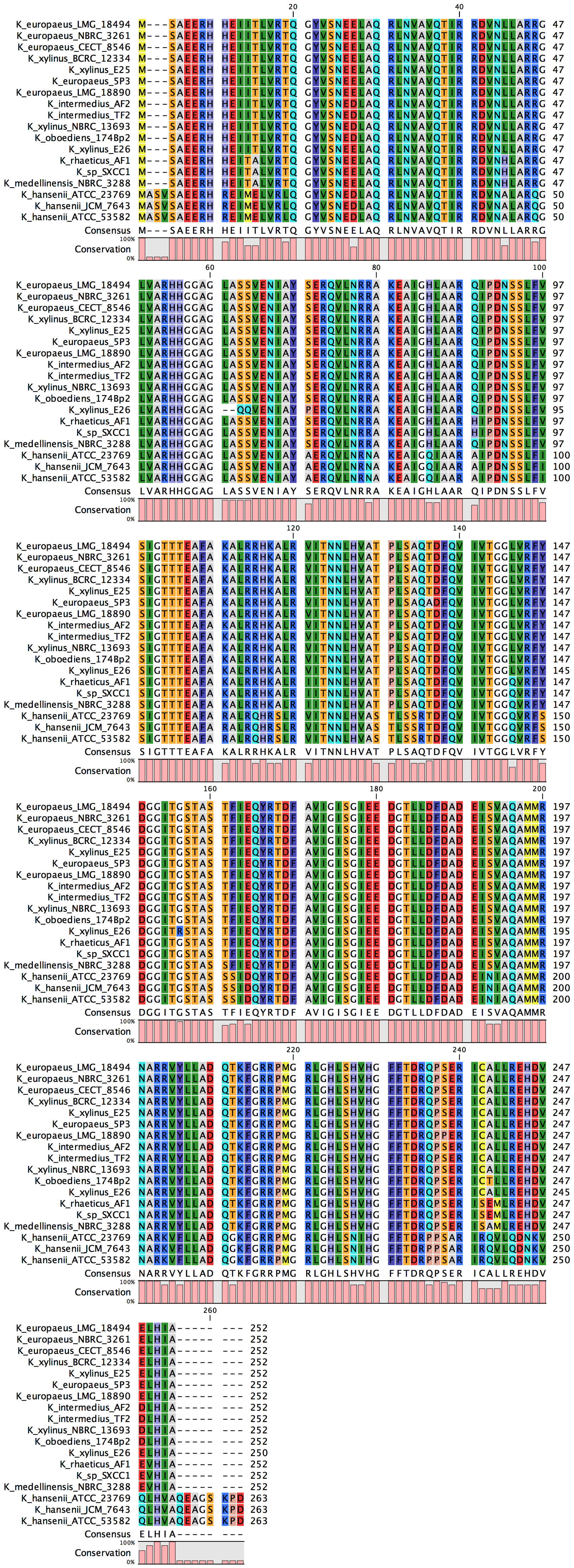


**Supplementary Figure 11**. Alignment of the GlpR amino acid sequence of the *Komagataeibacter* strains. Supplementary Figure generated using CLC Sequence Viewer.

**Supplementary Tables**

**Supplementary Table 1**. Predicted RepA proteins in the sequenced genomes based on sequence similarity with plasmid RepA proteins encoded by complete *Komagataeibacter* genomes.

| **Strain** | **No. of RepA homologs** | **RepA Acc. Number** | **Strain and plasmid of origin** | **Contig name** |
| --- | --- | --- | --- | --- |
| *K. xylinus* E26 | 2 | WP_081749530.1 | *K. xylinus* E25, pGX3 | NODE_74 |
|  |  | WP_078528626.1 | *K. nataicola* RZS01, pKNA04 | NODE_139 |
| *K. xylinus* BCRC 12334 | 2 | WP_081749530.1 | *K. xylinus* E25, pGX3 | NODE_79 |
|  |  | WP_078528626.1 | *K. nataicola,* RZS01,pKNA04 | NODE_132 |
| *K. hansenii* ATCC 53582 | 1 | WP_007284615.1 | *K. medellinensis* NBRC 3288, pGXY020 | NODE_20 |

**Supplementary Table 2**. Predicted CRISPR loci in the *Komagataeibacter* genomes.

| **Strain** | **No. of CRISPR loci** | **Length of repeats/spacers** | **No. of repeats** | **Seqeunces of the repeats** | **Sequences of the spacers** |
| --- | --- | --- | --- | --- | --- |
| *K. medellinensis* NBRC 3288 | 3 | 29/32; | 1: 8;  2: 16;  3: 14 | 1:GTGTTCCCCGCACGCGCGGGGATGAACCG,GTGTTCCCCGCACACGCGGGGATAAACCT;  2:CGGTTCAGCCTCGCGTATGCGGGGAACAC;  3:CCGTTTAGCCCTGCGGGTGCGGGGAACAC | 1:ATCACCGTTCTTGATTCGCCGCTGACCTCTGG,CGGGGATATGCCTGTATCTGGTGCAGAACAGG,GTGTTATCCTTCAGGATGCGGCAATCACGCCA,GCCGACAACGCGGCCATCCAGGCATCGTGAGC,CCTGCTTGCGGGCATGATAGGCGTCAAGGCGC,TTCAGGCGACCGCAAAGAAGCAGGCAGAAGAA,TAATCCACAATGTCCGGGTCGTTCTCAAAGGC;  2:GCTAATGTTAGAATAAGGTTAGCTCCCTCGAT,GATCATGTCATGAACGAAGCGCTGTGCCGGTC,AGACGTACGGAATGGGAATAAGTTTTTTGTTA,CCGACCGTAAAATCCAGACGGAAGGCCGCCGT,TCCTGCGTGAAGGTGGTCAGCTTGCCTTCCAG,CACAACGGCATGTCGGGCGTTGCCCCAGCCGT,AGATGCCGACCGATCTGCGTCAGTGCACCCAG,CACGATTGCACGGCGCTGGGCCGCAGATAGAA,TTCAAAACGAAGTTCGTAAAATAGCAGATTTC,CTATCCAGCCAGGTGGTGAAATCGTTGAACGC,TTAGTATGAGTGGGACAGTTGGGAAAACGACA,GCCAGACGGCGCACAGTATCATTGTTGGCGAT,ATGTTTCGAGTCCGCGGGAATGGCTGCGTGAG,CAGGGACCGGCGCTGTTCGTATGTCACAGCCT,CCGCGCACGTCACCATGCAATGCAGCATCGCC;  3:CACCGCCCAGTTGCTTGTCCGTGATACCCAGA,CACGACCACGTCGGGCAGCAGTTCCGGCACGA,GAGCCACACAACAATTACAACCGGCACTACTA,GTTCTGCGGCCAGTTTGCGGGATGATCGGGGT,CTCCAAATGGAGCCGACAAACCGCAATATTGC,AAATCATCGGAAATTTTTTCCTGTCCGGTCTG,AGGGTATGTGGCGAATGACGGGAACGGTGCAC,TCTGATATAGACGCGAGGACATTCCCCAGCGT,TCTGCCGCCACCTGTGTGGACACCCACGGGCG,GCCATATGAGCGGGTTGGACAAAGATACGCCG,GCATTGCCACGCATGGAAATGCCCGAATTGAT,TTCTTTGGCCCGATACCAGGTGAGCGTTCCGT,GAGGCACCTGTAGGTGCTGGCAAGTCGGTAAT |
| *K. hansenii* ATCC 23769 | 2 | 29/32 | 1: 6;  2: 4 | 1:CGGTTCATCCCCGCGTATGCGGGGAACAC;  2:GCCGACATCCCCGCGTGTGCGGGGAACAC,CGGTTCATCCCCGCGTGTGCGGGGAACAC | 1:AGGTCAGCAAACACGTTCGTGTTAAACTGGCT,TCAAGTTCTGCGTCTCGACGATCTTCTTCGTC,CGCGTAGTATAGGCCATCTCTCAGGGCGTGTT,CGCGCGCCGCGAATTCCTGCCAGTCCCGGCCA,CTGCGCAAGGCATGGCGCGTCACGGTTGACGG;  2:GGCCGGTCAACGTCCAGAAAAAACAGACGTGA,CGGCGCATTGAAGCGATGGAATAGGCGTAGCC,CGTTTTGAGACGTTCCACAACGGCTCTGTGAG |
| *K. hansenii* JCM 7643 | 2 | 29/32 | 1:6;  2: 4 | 1:CGGTTCATCCCCGCGTATGCGGGGAACAC  2:GCCGACATCCCCGCGTGTGCGGGGAACAC,CGGTTCATCCCCGCGTGTGCGGGGAACAC | 1:AGGTCAGCAAACACGTTCGTGTTAAACTGGCT,TCAAGTTCTGCGTCTCGACGATCTTCTTCGTC,CGCGTAGTATAGGCCATCTCTCAGGGCGTGTT,CGCGCGCCGCGAATTCCTGCCAGTCCCGGCCA,CTGCGCAAGGCATGGCGCGTCACGGTTGACGG;  2:GGCCGGTCAACGTCCAGAAAAAACAGACGTGA,CGGCGCATTGAAGCGATGGAATAGGCGTAGCC,CGTTTTGAGACGTTCCACAACGGCTCTGTGAG |
| *K. hansenii ATCC 53582* | 2 | 29/32 | 1: 17;  2: 20 | 1:GTGTTCCCCGCATACGCGGGGATGAACCG, GTGTTCCCCGCATGCCCTGTGCCATCGCC  2:GTGTTCCCCGCACACGCGGGGATGAACCG, GTGTTCCCCGCACACGCGGGGATGTCGGC | 1:AGAACCGGCTCCCGCTGCATCTTCCGGGGCAT,GCGCTGGCTCCTTCTTTCGCCCGTCCCGCAAC,ACGGACGAAAATTGTCACAAGCACATTCGGGC,CACTGCCACTGATGCGCTTATTCGATCGGGCG,GCGGCGTGGCTACGCTTGACGCCAGTGGGAAT,GCCCTGACGTTCCACGGTCCGACCGGCAGCAC,TAAAGAACATCAATTCCGATTTTATCGAAAGC,GCGGCGGCTGCTGCCACCGCTGCCTTGAACTC,CAGGAACTGGCCGGCGCTGCGCTTCAGGAGGC,ATACACAGCAAACGCTGATCCATCCAAATCCG,ACGATTGAATCCTTTTTTATACACTTGAGTAA,GGTTTAACAGTTTGTGATGTGACATAGCTGGT,GCGTCCCGTCCTCAACATCTGCGGATAATCCA,GGAGCCAAGTCAAGCTGTATTGGGGAATGTTG,GGCAGCGGGTCCGGCACGTCGCTGGTGCGTTA,TCGAACACGAGATCCCCCAACCCGACCTCGCG;  2:TGTTTCCTGCTCTTCCACATCCGCCGGGTCTT,CCCTACGTGCAGAACTGGCTGCGGACGCGGCA,GCTCTTTGCAGACCCGCAAATGGACGGACCAG,ATCCTGTGGCCCATCCTCAACAAGTTGGATCA,GCGATTTCCTCTTTGGCTTGAAGTTCGACTTT,GCGTGGGCCAACATCTCCGATACATCAATCGC,TGGGCACAGTCTACGCGCGGGACAACCTCGAC,CCCCTGAACCATTCCGTCACTTCATCCACGCT,ATCCTGCTCACAACCCACCAGCGCGTGCGGAT,AAACGCAATAGAACCATTTTTTGTGATGCGAA,GTAAAGGCGGCGAGTGGCTTCTATGACATTCT,TGCCACCAACACTGAACATGGTGCTGTCGCCG,CAGCGTGTCGATGCGCTGTCCTGCGCTGTTGA,GGCATATTTCGACATACCTGTGATGGTCTCAT,GCCATGCTCCAGAGGTTTTTTGCCTCCTGAAG,TCTGGCCTGACTGCCTTTGCGACCGCCGCCGG,GTGTCCGAGATCGTGCGCCAGCTTGCCGGTCG,GCGTGGTGTCGGGTATCTGGACTATCGATTCC,CATACATTACATGTGCGCACACACATGCACAC |
